# Extracellular matrix remodelling in dental pulp tissue of carious human teeth through the prism of single-cell RNA sequencing

**DOI:** 10.1038/s41368-023-00238-z

**Published:** 2023-08-02

**Authors:** Anamaria Balic, Dilara Perver, Pierfrancesco Pagella, Hubert Rehrauer, Bernd Stadlinger, Andreas E. Moor, Viola Vogel, Thimios A. Mitsiadis

**Affiliations:** 1 Orofacial Development and Regeneration, Institute of Oral Biology, Centre of Dental Medicine, University of Zurich, Zurich, Switzerland; 2 Department of Health Sciences and Technology, Institute of Translational Medicine, ETH Zurich, Zurich, Switzerland; 3 Functional Genomics Center Zurich, ETH Zurich and University of Zurich, Zurich, Switzerland; 4 Clinic of Cranio-Maxillofacial and Oral Surgery, University of Zurich, Zurich, Switzerland; 5 Department of Biosystems Science and Engineering, ETH Zurich, Basel, Switzerland

**Keywords:** Mechanisms of disease, Dental caries

## Abstract

Carious lesions are bacteria-caused destructions of the mineralised dental tissues, marked by the simultaneous activation of immune responses and regenerative events within the soft dental pulp tissue. While major molecular players in tooth decay have been uncovered during the past years, a detailed map of the molecular and cellular landscape of the diseased pulp is still missing. In this study we used single-cell RNA sequencing analysis, supplemented with immunostaining, to generate a comprehensive single-cell atlas of the pulp of carious human teeth. Our data demonstrated modifications in the various cell clusters within the pulp of carious teeth, such as immune cells, mesenchymal stem cells (MSC) and fibroblasts, when compared to the pulp of healthy human teeth. Active immune response in the carious pulp tissue is accompanied by specific changes in the fibroblast and MSC clusters. These changes include the upregulation of genes encoding extracellular matrix (ECM) components, including *COL1A1* and *Fibronectin (FN1)*, and the enrichment of the fibroblast cluster with myofibroblasts. The incremental changes in the ECM composition of carious pulp tissues were further confirmed by immunostaining analyses. Assessment of the Fibronectin fibres under mechanical strain conditions showed a significant tension reduction in carious pulp tissues, compared to the healthy ones. The present data demonstrate molecular, cellular and biomechanical alterations in the pulp of human carious teeth, indicative of extensive ECM remodelling, reminiscent of fibrosis observed in other organs. This comprehensive atlas of carious human teeth can facilitate future studies of dental pathologies and enable comparative analyses across diseased organs.

## Introduction

Carious lesions are bacteria-caused tooth-specific injuries, the outcome of which depends on a fine balance between inflammatory responses and the regenerative capability of dental pulp tissues.^1,2^ Although the molecular and cellular modifications in dental pulp tissues in response to carious lesions have been extensively studied over the last decades, a comprehensive analysis of these changes is still not well-established. Histomorphological analyses and gene expression studies have provided limited information on the effects of carious lesions on human dental pulp composition, molecular profile and cellular structure.

Bacterial invasion leads to the destruction of tooth mineralised matrices, enamel and dentin, further potentiated by activation of matrix metalooproteinases (MMPs), including MMP-2, MMP-9 and MMP20 released from the newly degraded dentin.^3^ Proinflammatory responses are triggered in the dental pulp, including the activation of signalling cascades that involve the NFΚB pathway and stimulate the expression of proinflammatory cytokines and chemokines, such as IL-1A, IL-1B, IL-6, IL-8 and TNFα.^4,5^ Consequently, immune cells, including dendritic cells and monocytes, are activated and recruited to the site of injury.^2,4,6^ These molecular and cellular cascades that constitute the immune response to the carious lesions have been extensively studied.^2,4^ Pivotal part of the response to carious lesions resides within the heterogeneous dental pulp, composed of various cell types, that differ in origin, function and degree of cell commitment and differentiation, and are embedded in a complex network of extracellular matrix (ECM).^7,8^ The most abundant cell type present in the dental pulp are the fibroblasts, which regulate extracellular matrix (ECM) formation and turnover, and display high functional and molecular diversity.^7,9^ Dental pulp also contains mesenchymal stem cells (MSCs) that participate in the regeneration and homoeostasis of the dental pulp tissue.^10,11^ Replenishment of the injured pulp tissue by MSCs mostly depends on released cytokines and chemoattractant molecules that coordinate cell migration through the also affected ECM pulp network.^10,12^

The ECM of the dental pulp is a loose network composed of molecules such as Collagen type I and type III and Fibronectin. Its overall composition and function in dental pulp tissues of healthy human teeth are extensively studied and understood.^8,13^ ECM provides physical support for spatial organisation of the dental pulp cells, and regulates various biological processes, including mineralisation.^13,14^ However, the effects of the carious insults on the biological and mechanical properties of the ECM are poorly understood. Generally, regeneration and/or repair upon injury of soft tissues is associated with ECM reorganisation and transient myofibroblast activity that leads to tissue functional restoration.^15,16^ Myofibroblasts share many molecular and functional similarities with fibroblasts and MSCs, and are essential for ECM turnover during the early stages of injury.^15^ However, the extent of ECM remodelling and the potential role of myofibroblasts in the carious pulp tissue is not well understood.

In this study, we used single-cell RNA sequencing (scRNAseq) analysis to unravel the cariogenic effects on the molecular and cellular composition of human dental pulp, by comparing the transcriptomes of healthy and carious human dental pulp tissues. Our data show significant changes in the dental pulp stromal cells and illustrate a vast remodelling of the ECM network resembling pathological organ fibrosis. Furthermore, we show important differences in the mechanical properties of ECM between healthy and carious human teeth. Collectively, our data provide the comprehensive single-cell RNA atlas of human carious teeth, and demonstrate the significance of ECM for tissue repair and regeneration upon injury.

## Results

### Molecular profile of dental pulp exposed to carious lesion

**Fig. 1:**
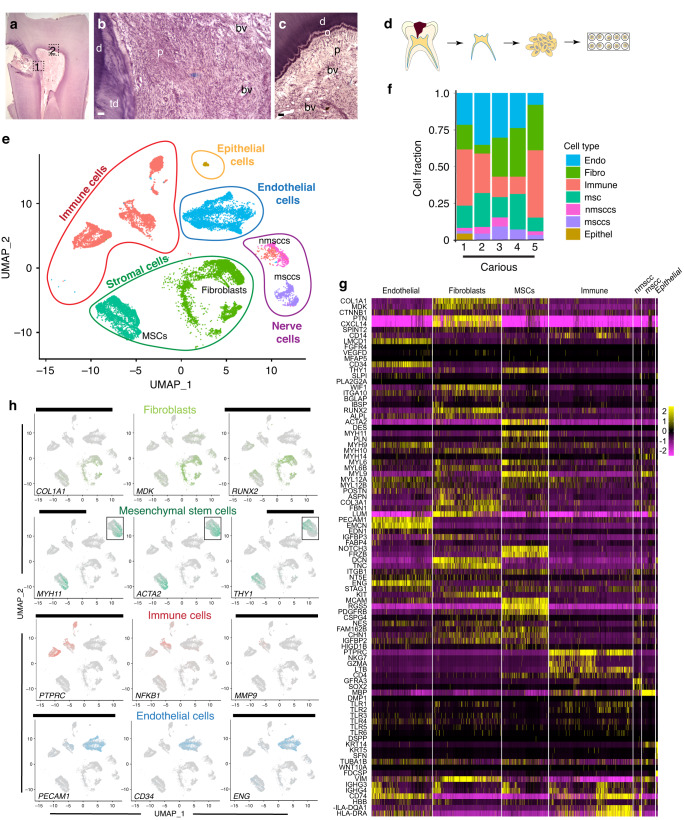
Morphological and molecular analysis of carious lesion. **a**–**c** Hematoxylin and Eosin (H&E) staining of a carious tooth. **a** Low magnification image showing carious lesion in the crown. **b**, **c** High-magnification images of the areas marked in **a** by a black dotted square 1 (**b**) and 2 (**c**). **d** Schematic representation of the single-cell RNA sequencing (scRNAseq) experimental setup, depicting the isolation and enzymatic processing of the dental pulp to a single-cell suspension processed for 10x genomics. **e** Colour-coded visualisation of cell clusters in UMAP plots: stromal cells (green; mesenchymal stem cells (MSCs) – dark green and fibroblasts – light green), immune cells (red), epithelial cells (orange), endothelial cells (blue) and nerve cells (purple; non-myelinating Schwann cells (nmsccs) – pink and myelinating Schwann cells (msccs) – violet). **f** Graphical representation of cell fractions per individual cluster, in each carious pulp sample. **g** Heatmap showing the expression of known markers for the respective cell types. **h** Distribution of the expression selected markers in the cells visualised in the UMAP space. d dentin, o odontoblasts, p pulp, td tertiary dentin, bv blood vessel. Scale bar represents 200 μm

**Fig. 2:**
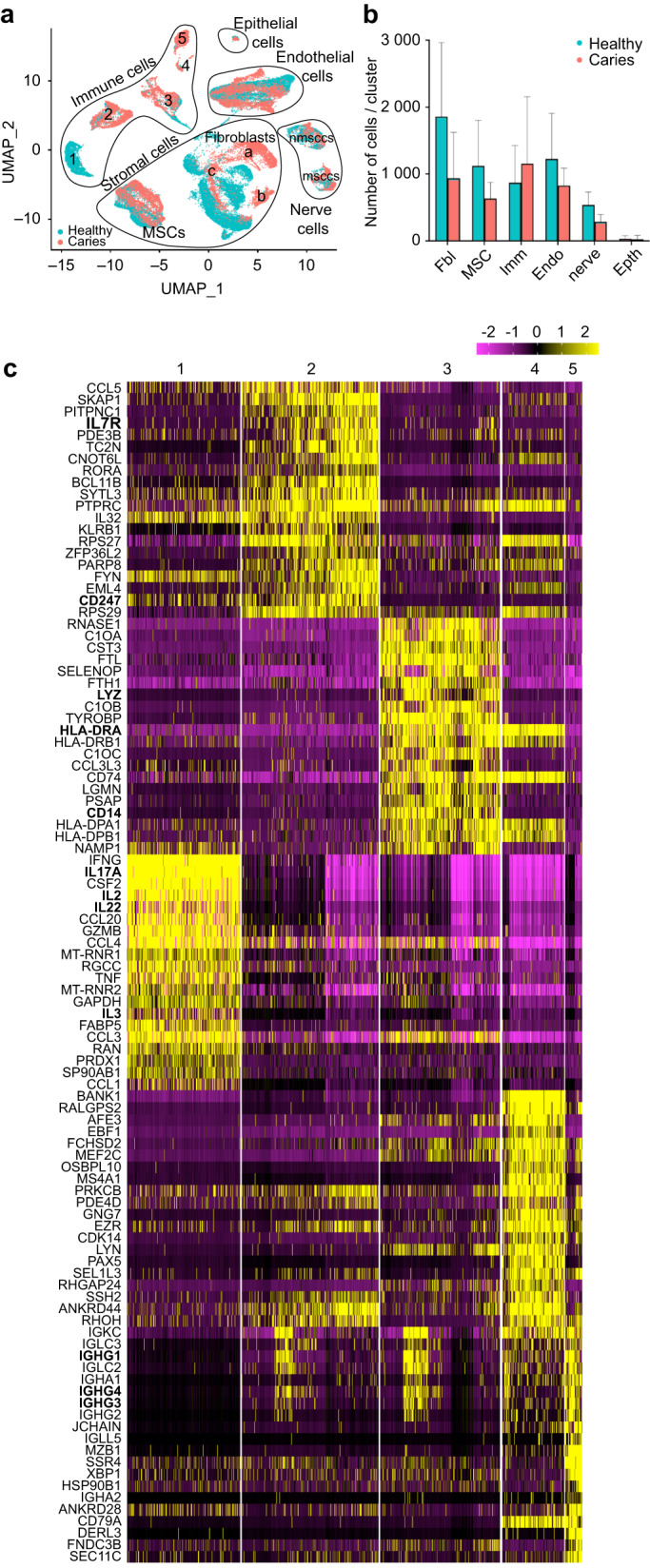
Comparative analysis of the transcriptome profile of carious and healthy pulp. **a** UMAP presentation of five major clusters (immune cells, stromal cells, epithelial cells, endothelial cells and nerve cells) in carious (red) and healthy (green) dental pulps. **b** Comparison of cell fractions in major clusters between the healthy (green) and carious (red) pulps. **c** Heatmap depicting the expression levels of top 20 marker genes for each of the five cell subclusters indicated in **a**. The genes were identified by Seurat

Caries are characterised by enamel and dentin destruction, and consequently changes in the dental pulp, that could lead to the secretion of tertiary dentin (Fig. 1a–c). There is a great variability in the course and outcome of carious lesion, and the determining factors are not fully understood. We aimed to characterise the molecular landscape of carious lesion that governs cellular behaviours and lesion progression, by performing single-cell RNA sequencing (scRNAseq) on dental pulp cells obtained from carious teeth (Fig. 1d, e). In total, 19,305 dental pulp cells from 5 patient samples were evaluated by unsupervised clustering in Seurat v4^17^ and visualised in UMAP (Uniform Manifold Approximation and Projection) space (Supplementary Table 1). Carious pulps contained 5 major clusters previously reported in healthy dental pulp^7^ (Fig. 1e, g). These clusters include stromal, immune, endothelial, nerve and epithelial cells (Fig. 2b), identified at variable sizes in all patient sample (Fig. 1f). Approximately 35% of analysed cells (7 836 cells) clustered within stromal cell cluster composed of fibroblast (4673 cells) and mesenchymal stem cell (MSC, 3163 cells) subclusters identified by *Collagen type I* (*COL1A1), Midkine (MDK)* and *RUNX Family Transcription Factor 2 (RUNX2)* expression (Fig. 1h, i) and *Frizzled Related Protein* (*FRZB)* expression (Fig. 1g), respectively. Furthermore, *Myosin Heavy Chain 11 (MYH11)* and *α-smooth muscle actin* (*ACTA2)* expression marked a distinct cell populations within MSC subcluster that were clearly distinguishable from the one marked by *THY1* expression (insets in Fig. 1h), as previously reported.^7^ Nearly 30% of total cells (5777 cells) constituted immune cell cluster identified by *PTPRC (CD45)* expression. This cluster was composed of T and B-lymphocytes, monocytes, macrophages and dendritic cells (Figs. 1e and 2). Further cell clusters included endothelial cells (4126 cells, or ~21%) identified by *Platelet And Endothelial Cell Adhesion Molecule 1 (PECAM-1), CD34* and *Endoglin (ENG)* expression (Fig. 1) and nerve cells, composed of non-myelinating (nmsccs) and myelinating (msccs) Schwann cells previously identified in the healthy pulp.^7^

We next compared data obtained from the pulp of carious teeth to the pulp of healthy teeth that we published previously^7^ (Fig. 2a). Quantitative analysis revealed no obvious change in the overall size of each cluster in terms of cell proportion between the two data sets (Fig. 2b), which could be attributed to high variability between the samples. Gross examination of differential gene expression showed that among the top 25 upregulated genes were genes involved in immune response, namely activation of B lymphocytes, phagocytosis and antigen recognition (Supplementary Table 2 and Supplementary Data 1). Hence, we first analysed the immune cell cluster.

**Fig. 3:**
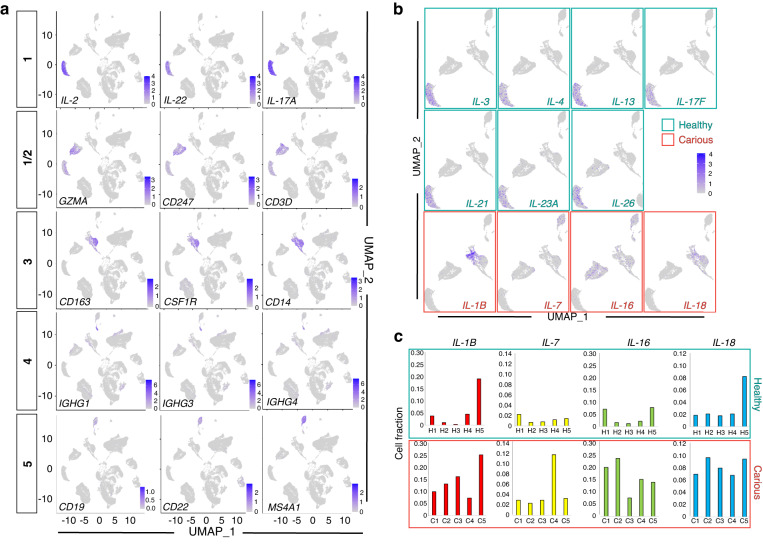
Comparative analysis of the immune cell cluster of carious and healthy pulp. **a** Feature UMAP plot representation of specific subclusters of immune cells. **b** UMAP visualisation of the distribution of *IL-3, IL-4, IL13, IL-17F, IL-21, IL-23A, IL-*26, IL-1B, IL-7, IL-16, and *IL-18* in the immune clusters of healthy and carious pulps. Green framed graphs illustrate IL genes expressed mainly in the healthy pulps, and red framed graphs illustrate IL genes expressed mainly in the carious pulps. **c** Fraction of cells expressing IL-1B, IL-7, IL-16 and IL-18 in the individual samples

UMAP visualisation revealed distinct sub-clustering of immune cells from carious pulps when compared to healthy pulp cells (Fig. 2a). While the immune cells of the healthy pulp were distributed among all the clusters, majority of these cells positioned within the subcluster 1 (Fig. 2a, c) marked by high levels of different Interleukins (IL), namely *IL-2, IL-3, IL-4, IL-13, IL-17A, IL-17F, IL-21, IL-23A* and *IL-26* expression (Figs. 2a, c, 3a, b). Quantitative analysis of these markers showed they were mainly absent in the carious pulp data set, that is marked by higher proportion of cells expressing *IL-1B, IL-7, IL-16* and *IL-18* (Fig. 3c, Supplementary Fig. 1a, b), albeit at variable levels between the carious samples (Fig. 3c). Interestingly, key marker genes for T-lymphocytes, namely *Granzyme A (GZMA), CD247* and *CD3D* (Fig. 3a), which were expressed in the subcluster 1, were also found in the subcluster 2. This suggests that subcluster 2 is composed mainly of T-lymphocytes.^18^ This is further supported by enrichment of this cluster for cells expressing *IL-7R* that is normally expressed at high levels in T-lymphocytes^19^ (Supplementary Fig. 2). Cluster 3 was marked by the exclusive expression of *CD163*, *Colony Stimulating Factor 1 Receptor (CSF1R)* and *CD14* (Fig. 3a), which identify monocyte cell lineage.^18^ Other markers, such as *CD83, HLA-DRA* and *Interferon Regulatory Factor 8 (IRF8)* (Supplementary Fig. 2), that mark subsets of monocyte, B-lymphocyte and dendritic cell lineages,^18^ were also expressed in the subcluster 3. Subcluster 4 was marked by exclusive expression of *IGHG1, IGHG3* and *IGHG4*, which marks memory and naïve B-cells which were detected in insignificant numbers in the healthy pulp data set (Fig. 3a and Supplementary Fig. 1c, Supplementary Data 2). Co-localisation of *CD83, HLA-DRA* and *IRF8* (Supplementary Fig. 2) with *CD19, CD22* and *Membrane Spanning 4-Domains A1 (MS4A1)* (Fig. 3a) in the subcluster 5 indicates that this cluster is composed mainly of B-lymphocytes. Collectively, this data demonstrate that immune cell lineages of the healthy pulp are all found in the carious pulp set, but their transcriptome profile is changed. Increased expression levels of specific Interleukin molecules and increased proportion of monocyte/macrophage, dendritic and B-lymphocyte lineages indicate activation of the specific immune and inflammatory responses to bacterial invasion.

**Fig. 4:**
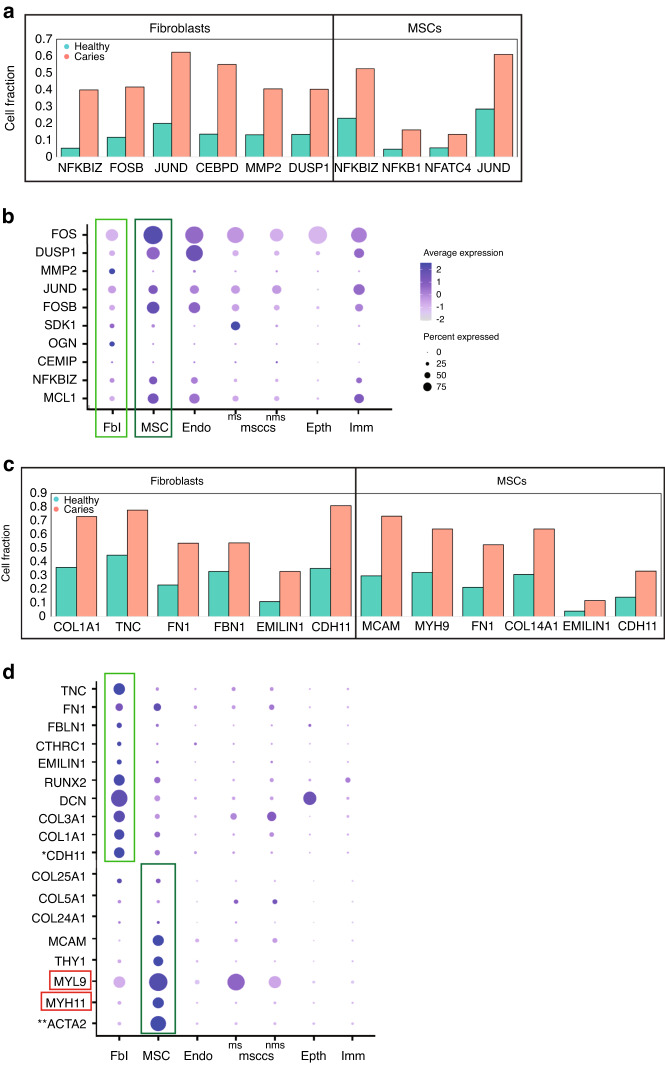
Comparative analysis of the stromal cluster of the carious and healthy dental pulps. **a** proportion of cells expressing targets of activated NFKB and TGFβ signalling in the specific stromal subclusters of healthy (green) and carious (red) dental pulps, namely fibroblast (left) and mesenchymal stem cell (MSC, right) subclusters. **b** Dot plot depiction of the expression parameters of selected genes, targets of activated NFKB and TGFβ signalling. **c** proportion of cells expressing ECM-related genes in the specific stromal subclusters of healthy (green) and carious (red) dental pulps, namely fibroblast (left) and MSC (right) subclusters. **d** Dot plot showing the expression parameters of selected ECM-related genes

Among the top 25 upregulated genes in the carious pulp set was *Serum Amyloid A1 (SAA1)* gene (Supplementary Table 2), an acute-phase protein produced in response to tissue injury, inflammation and cancer.^20^ This gene was specifically upregulated in the fibroblast population within the stromal cluster (Supplementary Table 2) that was marked by increased proportion of cells expressing genes associated with activated Transforming Growth Factor beta (TGFβ), Tumour Necrosis Factor (TNF) and *Nuclear Factor Kappa B* (NFκB) signalling pathways (Fig. 4a). In the fibroblast subcluster number of cells expressing *NFKB Inhibitor Zeta (NFKBIZ), FOSB, JUND, CCAAT Enhancer Binding Protein Delta (CEBPD)*, and others was increased more than fivefolds (Fig. 4a). Increased number of cells expressing genes targets of activated TGFβ, TNF and NFκB signalling pathways, like *NFKBIZ*, were also observed in the MSC subcluster (Fig. 4a). The expression levels of majority of these genes were also increased in both subclusters of the stromal compartment of carious pulps (Fig. 4b and Supplementary Data 3). NFKBIZ expression is activated by NFkB, establishing a negative feedback loop, as well as IL-1B.^21^ Similar to JUN and FOS genes, it is regulating immune response through interactions with Toll-like receptors (TLR).^21,22^ We detected a low number of cells expressing individual *Toll-like receptors (TLR)* in the fibroblast cluster of carious pulps. Approximately 5, 7 and 6.5% of cells expressed *TLR1, TLR3* and *TLR4*, respectively (Supplementary Table 3). Albeit low, this number was increased when compared to healthy pulps (Supplementary Table 3). Pulp fibroblasts reportedly also secrete various cytokines, which enables them to further modulate the inflammatory response of dental pulp.^1,5,23,24^ Within fibroblast subcluster of the pulp of carious teeth, we found approximately 9% of cells expressing *IL-7*, compared to almost 7% in the healthy pulps (Supplementary Fig. 2d and Data 2). Only a small fraction of cells (<0.3%) expressed *TNFα*, *IL-6* and *IL-18*, and cells expressing *IL-1* or *IL-8* were not detected in this cluster (Supplementary Data 2).

**Fig. 5:**
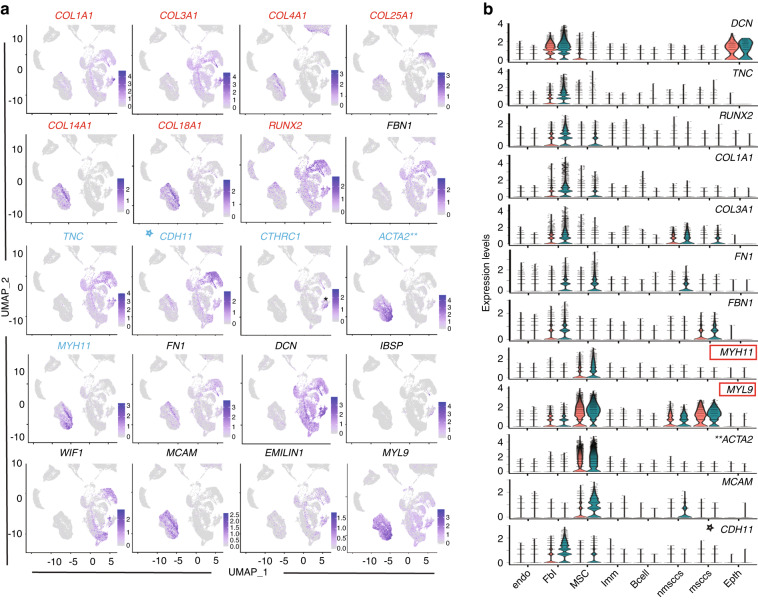
ECM-specific changes in the stromal cluster of the carious and healthy dental pulps. **a** Feature UMAP plot representation of the specific cell populations expressing ECM constituent genes (red and black) and myofibroblast-related genes (blue). **b** Violin plot demonstrating the expression levels of selected ECM-related genes

A more striking change in the fibroblast subcluster was the increase in the proportion of cells expressing various ECM-related molecules. There was ≥ 4-fold increase in the number of cells expressing *Osteoglycin (OGN)* (Fig. 4b), an ECM-associated proteoglycan associated with physiological processes like collagen fibrillogenesis, as well as pathological conditions, such as cancer and ectopic bone formation.^25^ The expression levels of *OGN* were also significantly increased (logFC 0.68, Supplementary Data 3). We also detected 15% more cells expressing *RUNX2*, *COL1A1, COL3A1* (Figs. 4c, d and 5a, b). In addition, expression levels of these markers increased for over 1.7 folds, compared to healthy pulp (Fig. 5b and Supplementary Data 3). Interestingly, the fraction of cells expressing *Integrin Binding Sialoprotein (IBSP)*, a marker of osteoblast/odontoblast differentiation,^26^ in the fibroblast cluster of carious pulps was ~5.4%. This is a 22.5-fold increase when compared to fraction of *IBSP+* cells in the fibroblast cluster from healthy pulps, where only 0.24% expressed this marker (Supplementary Data 2). Collectively, these data suggest the activation of the reparative processes the carious pulps.

Overall, the stromal cluster of carious pulps showed more than 1.5-fold increases in the expression levels, and the number of cells expressing different ECM-related molecules, including different Collagen types, including *COL1A1, Tenascin-C (TNC), Fibronectin (FN1)*, *Fibrillin 1 (FBN1), Elastin Microfibril Interfacer 1 (EMILIN1)*, etc. (Fig. 4c). Of these, the most interesting was a 2-fold increase in *TNC* expression that was accompanied by an increase from 45% of *TNC+* cells in healthy pulps to approximately 78% of *TNC+* cells in carious pulps (Figs. 4c, d and 5a, b). TNC regulates collagen-producing myofibroblasts, which transiently appear in response to injury in various tissues.^15,27^ Myofibroblasts are marked by expression of *ACTA2* and *Cadherin 11* (*CDH11)*, among others.^15,28^ Analysis of the myofibroblast-related genes demonstrated distinctly distributed qualitative and quantitative changes between the fibroblast and MSC subclusters. In the fibroblast subcluster, the expression levels and the proportion of cells expressing *ACTA2* was similar between the healthy and carious pulp data sets (two asterisks in Figs. 4c, d and 5a, b and Supplementary Data 2 and 3). In contrast, there was over 2-fold increase in *CDH11* expression levels, as well as increase in the number of *CDH11* expressing cells (star in Figs. 4c, d and 5a, b and Supplementary Data 2 and 3). Approximately 22% of fibroblasts from carious pulps expressed another known marker of myofibroblasts, *Collagen Triple Helix Repeat Containing 1 (CTHRC1)* that identifies myofibroblasts in the fibrotic lungs and heart.^29,30^ This was a 2-fold increase, compared to healthy pulp, where only 11% of these cells were detected in the fibroblast subcluster (Supplementary Data 2). Interestingly, *CTHRC1+* cells were abundant in the fibroblast subcluster from one carious pulp sample when compared to other samples, both carious and healthy (asterisk in Fig. 5a). Recent transcriptomics analyses of the fibrotic skin proposed Secreted Frizzled Related Protein (SFRP) 2 and 4 as a new myofibroblast markers.^31^ We also observed increase in the proportion of *SFRP2*+ cells (~0.9% in carious pulps vs. ~0.3% in healthy pulps) and *SFRP4*+ cells (~8% in carious pulps vs. ~4% in healthy pulps) in the fibroblast subcluster of carious pulps (Supplementary Data 2).

The MSC subcluster was marked by more than twofold increase in the number of cells expressing genes related to ECM organisation and remodelling, angiogenesis and vasculature development and wound healing, among others (Figs. 4c, d and 5). These include increased fractions of *Melanoma Cell Adhesion Molecule (MCAM), Myosin Heavy Chain 9 (MYH9), FN1* and *COL14A1* expressing cells (Figs. 4c, d, 5 and Supplementary Data 2), that constituted more than 50% of the MSC subcluster in the carious pulps. In addition, significantly increased *ACTA2* expression marked approximately 75% of cells in carious pulps, compared to ~ 63% in the healthy pulps (two asterisks in Fig. 5, Supplementary Data 2 and 3, Supplementary Fig. 3). Expression levels of other genes associated with myofibroblast phenotype, such as *MYH11*^32^ and *Myosin Light Chain 9 (MYL9)*,^33^ were also increased in the MSC subcluster (red boxes in Figs. 4d and 5a), but the proportion of cells expressing these markers was comparable between the carious and healthy pulp cells (Supplementary Data 2).

It is worth nothing that the changes in the MSC subcluster were accompanied by changes in the fraction of cells expressing *PECAM-1* (0.801 in carious pulps vs. 0.56 in healthy pulps) (Supplementary Fig. 3, and Supplementary Data 2), as well as *PECAM-1* expression levels (logFC 0.59, Supplementary Data 3). In addition, we also observed increased fraction of cells expressing genes related to cell motility and adhesion (*IGF1R, FBLN1, CXCL12, FN1*, etc.*)*,^34–36^ and cell apoptosis (*FOXO3, MCL1*, etc.)^37,38^ in both fibroblast and MSC subclusters (Supplementary Data 2 and 3).

### Altered ECM composition upon carious lesion

**Fig. 6:**
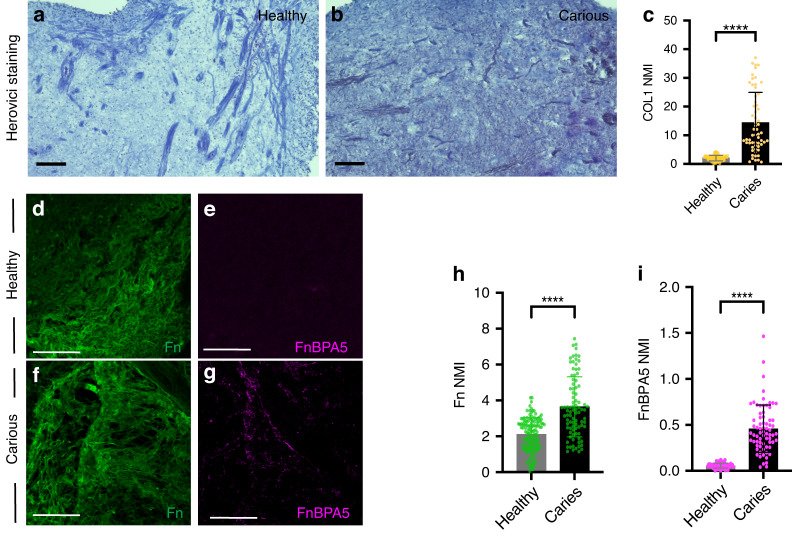
Analysis of extracellular matrix (ECM) changes in the carious pulps. **a**–**c** Herovici histological staining (**a**, **b**), and the quantification of COL1A1 fluorescent immunostaining intensity (**c**) in the healthy (**a**) and carious (**b**) pulp cryosections. **d**–**i** Analysis of the Fibronectin tension state. Representative confocal microscopy images of cryosections from healthy (**d**, **e**) and carious (**f**, **g**) pulps immunostained for total Fibronectin (green, **d**, **f**) and tension-sensitive bacterial peptide FnBPA5 (magenta, **e**, **g**). **h**, **i** Quantification of total Fibronectin (**h**) and FnBPA5 (**i**) fluorescent intensity, with data presented as mean ± S.D., ****P< 0.000 1. Scale bars represent 100 μm

**Fig. 7:**
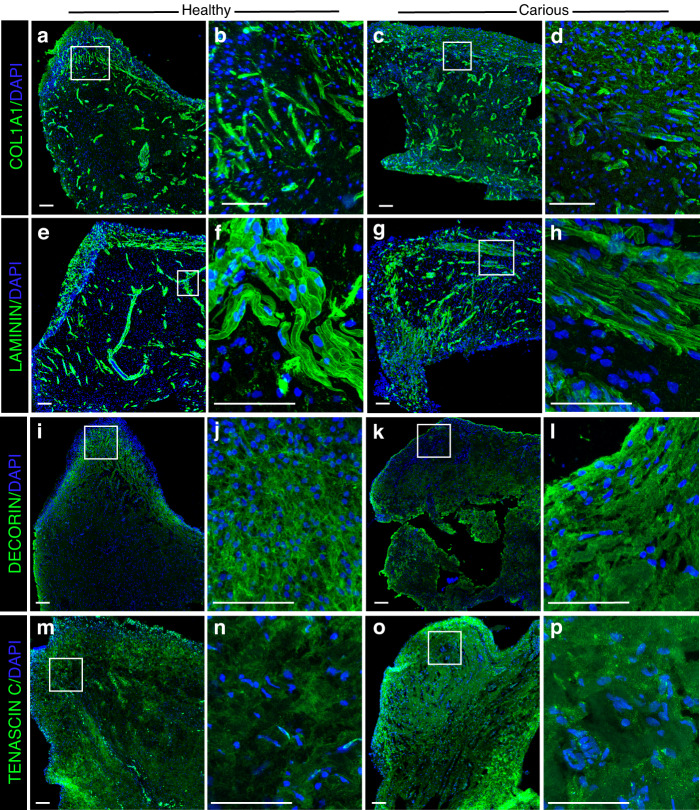
Extracellular matrix (ECM) changes in the carious pulps. **d**–**k** Immunostaining analysis of COL1A1 (**a**–**d**), LAMININ (**e**–**h**), DECORIN (**i**–**l**) and Tenascin-C (TNC) (**m**–**p**) protein expression (green) in the cryosections of healthy (**a**, **b**, **e**, **f**, **I**, **j**, **m**, **n**) and carious (**c**, **d**, **g**, **h**, **k**, **l**, **o**, **p**) pulps. DAPI staining (blue) identifies the nuclei. (**b**, **d**, **f**, **h**, **j**, **l**, **n**, **p**) are higher magnification images of the areas marked by the white square in **a**, **c**, **e**, **g**, **I**, **k**, **m**, **o**. Scale bars represent 100 μm

Transcriptional changes in the expression of ECM protein components suggest potential ECM remodelling and changes in the biomechanical properties of the ECM. Herovici staining of histological sections labels Collagen deposits, and it demonstrated more intense staining in the carious pulps (Fig. 6a, b), which was also confirmed by quantification of COL1A1 immunoreactivity that was increased in the carious pulps (Figs. 6, 7a–d). Similarly, immunoreactivity towards other constituents of dental pulp ECM, such as Laminin (Fig. 7e–h), Decorin (Fig. 7i–l), Tenascin-C (Fig. 7m–p) and Fibronectin (Fig. 6d, f, h) was also increased in carious dental pulp. These data further confirmed our hypothesis of the active ECM remodelling induced by carious lesion. To determine the extent of these changes and their effect on the biomechanical properties of the ECM, we compared the tensional state of Fibronectin in the healthy and carious dental pulps (Fig. 6e, g, i). Healthy tissues show high tension of Fibronectin fibres, as evidences by a lack of binding of the Fibronectin tension probe FnBPA5 that specifically binds to N-terminal Fibronectin fragments.^39^ FnBPA5 binding to N-terminal Fibronectin fragments depends on the tensional state of Fibronectin fibres, as their stretching destroys the peptide’s multivalent binding sites.^40^ In line with this, healthy dental pulp demonstrated no FnBPA5 staining (Fig. 6e). In contrast, dental pulp from carious teeth demonstrated relaxed Fibronectin fibres as evidenced by increased FnBPA5 staining (Fig. 6g, i). These data demonstrated changes in the tension of ECM fibres that could affect the reparative processes triggered by carious lesion, as well as the cellular behaviours that determine outcome of carious lesion.

## Discussion

The clinical presentation and outcome of dental caries depend on complex biological, environmental, behavioural and patient-specific factors that are poorly understood.^2^ Various studies have identified individual cellular and molecular changes in carious dental pulp tissues, but the comprehensive analysis of key mechanisms which regulate the course and outcome of this disease are still poorly understood. In this study, we provide a comprehensive atlas of cellular and molecular changes occurring in carious dental pulps at unprecedented resolution. These scRNAseq analyses show that carious lesion leads to activation of specific immune responses, generated by activated monocyte/macrophage and dendritic cells and marked by increased levels of cytokines such as IL-1B, IL-7, IL-16 and IL-18, that is accompanied by unique molecular and structural changes in the stromal pulp compartment marked by upregulation of genes targeted by active NFKB and TGFβ signalling, and a shift in transcriptome signature that resembles molecular changes observed during pro-fibrotic events in various organs. These changes include increases in the expression of myofibroblast-related genes, such as *ACTA2, CDH11, CTHRC1* and others, as well as various ECM protein genes, including *COL1A1, COL3A1, FN1, TNC* and others, which contribute to ECM remodelling and modifications of mechanical properties.

The first line of defence against caries are odontoblasts that play a role in pathogen recognition, activation of the innate immune response and secretion of reactionary tertiary dentin to resolve the insult.^41^ However, in our data set we have recovered a very small number of odontoblasts, which prevented any meaningful analysis of this cell population. In the remaining pulp tissue we have observed activation of TGFβ, TNF and NFκB signalling pathways, as previously reported,^1,42,43^ accompanied by a specific cytokine expression profile. Published RT-qPCR analyses of human pulp tissue exposed to caries report dissimilar cytokine expression profiles, including upregulation of IFN-γ in the majority of tested samples occasionally associated with increased IL-4 and/or IL-10 depending on the depth of the carious lesion;^44^ increased levels of IL-6, IL-8 and IL-18, but not IL-1α and IL-1β in carious pulps;^45^ upregulated TNF-α, IL-1β, IL-6 and IL-8^46^ or moderate upregulation of IL-1α, IL-10, IL-13 and IL-17C.^47^ These discrepancies are likely a reflexion of differences in the bacterial pathogen that initiated caries, diversity of bacterial pathogens associated with secondary bacterial infection and caries progression, as well as the extent/depth and the duration of the carious lesion between different samples.^44,46^ Furthermore, these studies indicated complex changes in the cytokine expression profile that affect both proinflammatory and anti-inflammatory cytokines. Likewise, our data set revealed distinct cytokine profile, namely increased number of cells expressing IL-1β, IL-7, IL-16 and IL-18 and low to absent numbers of cells expressing *IL-2, IL-3, IL-4, IL-13, IL-17A, IL-17F, IL-21, IL-23A* and *IL-26*. Also, similar to published studies, our data also showed high variability between individual carious and healthy samples. The majority of these molecules is produced by immune cells, except for the IL-7 that is mainly produced by stromal cells. We have observed a moderately increased proportion of cells expressing *IL-7* in the fibroblast cluster, but the most striking increase was the presence of *IL-7*-expressing cells in the subcluster 5 of the immune cells that was characterised as a B-cell subcluster. The available databases *(Human Protein Atlas proteinatlas.org)* also show *IL-7* mRNA expression in a similar population of immune cells.

Whether the observed cytokine profile reflects the response to specific bacterial pathogens remains unanswered, since the microbial and pathogen status of the caries in our samples is unknown. Another possibility is that it reflects the qualitative and quantitative changes of various cell subpopulations within the immune cell cluster. While quantitative analyses of the cellular profile within a sample are limited in scRNAseq data, all the major immune cell populations were present in the carious pulp samples. However, the transcriptome profile of immune cell subsets in the carious pulps was altered from that observed in the healthy pulps, likely reflecting an activation of the immune response to the bacterial infection. Namely, we observed the activation of the dendritic cells and macrophages, that participate in the initial stages of immune response.^6^

While the complex changes observed in the immune cell cluster are interesting, we were more intrigued by the changes observed in the stromal compartment, in which mild carious lesions induced the expression of genes encoding for ECM constituents, namely various Collagens, including COL1 and COL3 as well as FN1, DCN, TNC and others, which are the main constituents of pulpal ECM.^8,13^ An increased number of cells expressing COL1, as well as other genes related to osteoblast/odontoblast differentiation, such as RUNX2 and IBSP, indicates enhanced, ongoing repair in the carious pulp. Interestingly, the observed changes in the ECM composition also share similarities with the fibrotic changes observed in various organs.^48^ One of the interesting changes was an increased number of cells expressing TNC, an ECM glycoprotein involved in physiological and pathological ECM remodelling and repair. Increased TNC expression is observed in inflammation, organ fibrosis,^27,49,50^ and in cancer.^51^ Weak and restricted TNC expression in the majority of the healthy adult tissues is transiently induced during wound healing and tissue remodelling, but in pathological conditions, such as fibrosis and cancer, it persists.^52^ Among the TNC transcription regulators is TGFβ signalling,^53^ which is also activated in our carious pulps that demonstrate increased expression levels of TGFβ-1, TGFβ-2 and SMAD3, and a higher proportion of cells expressing these genes. Furthermore, studies on heart and skin tissues showed that TNC enhances the myofibroblast phenotype, via TGFβ signalling or through TRL-4, respectively.^49,54^ The increased number of cells expressing myofibroblast markers found in our carious pulp samples, is consistent with these findings.

Myofibroblasts share features reminiscent of both smooth muscle cells and fibroblasts and are important players in fibrotic disorders.^15^ While a small proportion of carious pulp myofibroblast-like cells share some molecular similarity with the myofibroblast cells of the fibrotic skin tissue that express *SPRF2* and *SPRF4*,^31^ our data set indicates more similarity with myofibroblast populations in cardiac and lung fibrotic tissues that are marked by *CDH11* and *ACTA2* expression. Our analyses show that cells expressing these markers constitute a significant proportion of the healthy pulp, as well. This could be attributed to the ageing-related changes normally observed in healthy pulps, which include decreased volume and increased fibrous tissue density.^55^ Furthermore, our analysis revealed the presence of two distinct myofibroblast-like cell populations in the carious stromal compartment, namely *CDH11* and *CTHRC1* expressing cells in the fibroblast subcluster, and *ACTA2, MYH11* and *MYL9* expressing cells in the MSC subcluster. Whether there is a developmental hierarchy between the two myofibroblast-like populations remains unclear. The high variability between the different pulp samples hampered further analyses and lineage modelling that could provide a more conclusive answer to this question. The high variability among the samples could be attributed to several factors, including the genetic variability between each individual, and other biological differences, including the age, tooth type, microbial cause, as well as the extent of carious lesion. In this study, we aimed to analyse the early carious lesion, which we assessed based on the robust and often unreliable evaluation of the clinical presentation of the caries, namely the extent of the damage in the visible portion of the tooth.

Increased levels of relaxed Fibronectin further indicated enhanced fibrotic changes that accompany pathologically impacted ECM found also in pulp tissues of carious teeth.^39^ Studies in humans and in animal models have shown that Fibronectin plays an essential role during early development, but also in fibrosis.^56–58^ Fibronectin regulates different aspects of ECM biogenesis and composition,^59^ and can contribute to myofibroblast activation.^60^ Increased levels of total Fibronectin protein in the carious dental pulps were accompanied by the abundance of relaxed Fibronectin identified by FnBPA5. Increased amounts of relaxed Fibronectin have been previously reported in the cancer stroma in the proximity to myofibroblasts.^39^ Fibronectin regulates cell adhesion and motility, which determine the speed of wound healing, as well as the invasiveness of cancer cells.^61,62^ Our scRNAseq data show activation of molecular regulators of cell contractility and motility in carious dental pulps, but it is unclear whether they are a direct result of changes in the Fibronectin tension status and ECM remodelling.

The changes in the composition and the biomechanical properties of the pulp ECM induced by carious lesions are similar to those observed in pathological fibrotic tissues of other organs, such as lungs^29^ and heart.^27^ One of the surprising findings was the relatively high number of myofibroblast-like cells and TNC-expressing cells in healthy pulps. Unlike other soft tissues, dental pulp is contained within the mineralised dentin, which significantly diminishes the stress from physical and mechanical forces of the surrounding tissues.^13^ One explanation could be that these changes relate to physiological, aging-triggered fibrotic changes that are regularly observed in the healthy dental pulp. These changes include diminished cellularity of dental pulp and increased amount of collagen, subsequently leading to remodelling of pulp tissue to adapt to narrowing of the pulp chamber.^63^ Thus, it can be postulated that the onset of aging in the healthy dental pulp might be associated with spontaneous activation of TNC and myofibroblast phenotype. Therefore, dental pulp could be a unique model for studying myofibroblast cells and the onset and molecular regulation of tissue fibrosis under homoeostatic conditions, and in the absence of tissue injury or disease.

Collectively, our study provides a remarkable insight into the carious-induced molecular changes in the human dental pulp, that generate a specific cellular and extracellular pulp tissue configuration. A pilot scRNAseq analysis of human dental pulp cells from carious teeth has been published recently.^64^ However, several shortcomings exist that hinder the interpretation of these results. Firstly, comparative analyses between healthy and carious teeth were performed on a low number of cells; 890 pulp cells from one healthy tooth and 5.692 pulp cells from three carious teeth. Next, the carious data set was generated from teeth also including deep carious lesions, which regularly induce apoptotic and necrotic events that compromise the quality and readout of the results.^1,41^ In this pilot study, pulp cells from one healthy tooth (890 cells) and from one tooth with enamel carious lesion (2.015 cells) were pooled together and compared to pulp cells from two teeth with deep caries (3.677 cells). This uncommon and unorthodox comparative analyses are based on a statistically irrelevant number of samples. In contrast,^64^ our results are based on a significantly higher number of dental pulp cells (19.305 cells) acquired from 5 teeth with mild caries that does not affect dental pulp tissue vitality, and compared to our previously obtained data from 5 healthy human teeth (28,763 cells).^7^ Moreover, the computational analyses in our study are further supported by substantial validation data, which are missing in the pilot study.^64^ Therefore, it is obvious that the present results are thorough, trustful, well-executed and clearly indicate the molecular and cellular variability of dental pulp tissues selected from various individuals.^13^ The present data stress the necessity for accurate aetiology and classification of caries in order to develop innovative therapeutic tools.^65^ Cellular and molecular modifications in dental pulp tissues occurring upon carious insults resemble and reflect alterations in other pathological tissues. Hence, dental pulp could represent a valuable model to study pathologies linked to soft tissues, including fibrosis.

## Materials and methods

### Human subjects

The procedure for the collection of anonymized human dental pulp and periodontal cells at the Centre of Dental Medicine was approved by the Cantonal Ethics Commission of Zurich (reference number 2012-0588) and the patients gave their written informed consent. Samples were obtained in fully anonymized form from patients of 18–35 years of age. Tooth extractions were performed by professional dentists at the Oral Surgery department of the Centre of Dental Medicine of the University of Zurich. Evaluation of the health status of the tooth was done post-extraction, upon direct observation of the specimen. Teeth with mild carious lesions affecting the crown, that conserve dental pulp vitality (no apparent necrotic pulp tissue) were selected for the study. Teeth showing extensive or deep caries, root caries and pulp necrotic tissue (upon tooth cracking and collection of the pulp tissue) were excluded from the study. The total of five carious human teeth comprised of one lower premolar, two lower and two upper wisdom teeth were used. All procedures were performed in accordance with the current guidelines.

### Cell isolation and single-cell transcriptomic analyses

#### Isolation of cells from the dental pulp

Teeth were collected immediately after extraction and placed in sterile 0.9% NaCl, on ice. Dental pulp was obtained from carious teeth following the protocol we previously applied for the isolation of healthy human pulp tissues.^66^ Briefly, each tooth was cracked with a press, and carefully opened with forceps. Isolated dental pulp was placed in a Petri dish filled with cold HBSS and minced into smaller pieces (<2 mm diameter) for easier dissociation. The dental pulp pieces were transferred to falcon tubes and dissociated using 5 U/mL Collagenase P (11 213 873 001, Sigma-Aldrich, Buchs, Switzerland) in PBS for 40 min at 37 °C. After incubation in the enzymatic solution, tissue pieces were further mechanically disaggregated by pipetting and centrifuged at 300 × *g* at 4 °C for 10 min. Cell pellet was resuspended in HBSS containing 0.002% Bovine Serum Albumin (BSA; 0163.2, Roth AG, Arlesheim, Switzerland) and filtered through a 70 μm cell strainer.

#### Single-cell RNA sequencing (scRNAseq) using 10X Genomics platform

The concentration of cells in the prepared dental pulp single-cell suspensions was adjusted to 1 000 cells per μL. Single-cell library was generated for each dental pulp sample using a droplet method, namely the Chromium System from 10× Genomics, Inc. (Pleasanton, CA, USA) and a Single-Cell 3′ v2 or v3 Reagent Kit (10× Genomics, Inc.) according to the manufacturer’s instructions. In each sample, 10 000 cells were loaded into the 10X Chromium controller and the resulting libraries were sequenced in an Illumina NovaSeq sequencer according to 10X Genomics recommendations (paired end reads, R1 = 26, i7 = 8, R2 = 98) to a depth of 50,000 reads per cell.

Individual cell data were filtered so that every cell with >20% of mitochondrial genes was excluded. From the remainder of the cells, every cell that expressed <200 genes, or 25 000 UMI was excluded.

The gene expression count matrix was computed using Cellranger v7. All data analysis was performed using Seurat v4^67^ and R version 4.2. Clusters were visualised using uniform manifold approximation and projection (UMAP) (McInnes et al., 2018). Samples were initially analysed separately, and data was scaled and transformed using SCTransform^68^ for variance stabilisation. The joint analysis of all samples was performed by integrating data using Seurat merge function. No additional data manipulation was performed. All statistical analysis of differential expression was performed on unintegrated data. Differential expression analysis was performed using the Wilcoxon rank sum test. All p values reported were adjusted for multiple comparisons using the Bonferroni correction.

#### GO analysis

To characterise the molecular changes found in scRNAseq data set, and to put them in a context of biological, cellular and molecular processes, we used g:Profiler, a simple user-friendly web interface with powerful visualisations.^69^

### Tissue processing and analysis

#### Tissue processing and embedding

Healthy and carious human dental pulps were fixed with 4% paraformaldehyde (PFA) at 4 °C overnight, immediately following the isolation. The following day, tissue was immersed in 30% Sucrose and kept at 4 °C until sinking. Sucrose-infused tissue was then transferred to Tissue-Tek O.C.T. Compound (4583, Sakura, Alphen aan den Rijn, Netherlands), snap frozen and serially cryosectioned in 10 μm thick sections.

#### Herovici staining

Young and mature COL1 were differentiated by Herovici staining performed on tissue cryosections washed with PBS, following kit instructions (18432, MORPHISTO GmbH, Offenbach am Main, Germany). The stained slides were gradually dehydrated and mounted with Eukitt Quick-hardening mounting medium (03989, Sigma-Aldrich).

#### Immunostainings

Cryosections of human dental pulps were washed with PBS, incubated with blocking solution (1%BSA, 10% donkey serum, 0.5% Triton in PBS) for 45 min at room temperature, followed by incubation with the primary antibody at +4 °C, overnight. Primary antibodies used were as follows: ACTA2 (αSMA, ab5694, 1:200), COL1A1 (ab34710, 1:100), COL3A1 (ab6310, 1:200), CD31 (PECAM, ab9498, 1:100), LAMININ (ab11575, 1:100), DECORIN (ab175404, 1:100), TENASCIN C (LS-B8789/74286, 1:50). After incubation with the primary antibody, sections were extensively washed and incubated with appropriate secondary antibody (1:500) for 45 min at room temperature. The secondary antibodies used include donkey anti-rabbit (AF488, A32790, Invitrogen) and donkey anti-mouse (AF647, A31571, Invitrogen). After incubation, slides were washed with PBS, mounted using Vectashield with DAPI, and imaged with Leica STELLARIS 5 automated upright confocal laser scanning microscope equipped with HC PL APO CS2 20x/0.75 NA Air and HC PL APO CS2 63x/1.3NA Glycerol objectives.

### Analysis of the Fibronectin fibres’ tension state

#### Immunostaining for Fibronectin, FnBPA5 and COL1A1

Tooth pulp cryosections were stained with Fibronectin and FnBPA5 probe as previously described.^39^ Briefly, thawed sections were washed with ice-cold 1xDPBS and blocked with 0.3 mol·L^-1^ glycine animal-free blocking buffer (Animal-Free Blocker® and Diluent, R.T.U. SP-5035-100) at room temperature for 30 min. Sections were then incubated with 1 μg·mL^-1^ Cy5-FnBPA5 or Cy5-labelled scrambled-FnBPA5 (scrambled-FnBPA5) for 1 h, followed by three 5 min washes with Dulbecco’s phosphate-buffered saline (DPBS), second blocking with the blocking buffer and incubation with polyclonal rabbit anti-Fibronectin (ab23750, Abcam) and monoclonal rat anti-Collagen I (7025, Chondrex) antibodies, overnight at 4 °C. The following day samples were extensively washed with DPBS before 1 h incubation with secondary antibodies and 2 μg/ml DAPI at room temperature. The secondary antibodies used were goat anti-rabbit (AF488, A11034, Invitrogen) and goat anti-rat (AF546, A11081, Invitrogen) antibodies. Following extensive washes after incubation with the secondary antibody, the samples were mounted using DAKO Fluorescence mounting medium (DAKO, Denmark) and imaged.

#### Confocal laser scanning microscopy (CLSM) Imaging

Stained tissue sections were imaged either as a whole section overview (tile-image), or as a randomly chosen high-magnification images. Tile images were acquired as z-stacks using a Leica SP8 confocal microscope with HC PL APO CS2 ×20/0.75 Air objective with 0.75 NA, and stitched together using the Mosaic Merge stitching function of LAS X software. DAPI, Fibronectin, Collagen I and FnBPA5 channels were imaged with 512 × 512-pixel resolution, and the laser power was kept constant. For high-magnification images HC PL APO CS2 63x/1.NA Oil objective was used.

#### Image analysis

Quantification analysis of the fluorescent intensity was performed using Fiji software. Control tissues incubated with Cy5-labelled scrambled peptides and secondary antibodies were used to determine the threshold values for each channel. Quantification was performed in randomly selected areas (50–100 μm × 50–100 μm) of tile-images, and on high-magnification images. In each area/image quantified, the individual signal intensity above the threshold was evaluated in every virtual section (*n* ≥ 6/z-stack). The FnBPA5-Cy5 and Fn-AF488 intensity ratios were also determined in every virtual section. The collected intensity values for each signal were normalised to DAPI mean intensity. Data were collected as experimental triplicates from 2 healthy and 2 carious pulp tissue samples.

#### Statistical analysis

Statistical analysis was performed in Graph Pad Prism (version 9.4.1) using unpaired student’s *t* test with Welch’s correction for *P* < 0.05.

## Supplementary information

Supplemental Tables

Supplemental Data 1

Supplemental Figure 1

Supplemental Figure 2

Supplemental Figure 3

Supplemental Data 2

Supplemental Data 3

Supplemental Data 3
