# Supplementary material for: Extracellular matrix remodelling in dental pulp tissue of carious human teeth through the prism of single-cell RNA sequencing
Source: Int J Oral Sci. 2023 Aug 2;15:30. doi: 10.1038/s41368-023-00238-z (PMC10397277; doi:10.1038/s41368-023-00238-z)
Supplement: Supplementary file 1 — Supplemental Tables [file 41368_2023_238_MOESM1_ESM.docx]

**Supplementary Table 1. Number of cells analysed by scRNAseq in each carious pulp sample.**

| **Sample** | **Cell number** |
| --- | --- |
| 1 | 3,364 |
| 2 | 2,645 |
| 3 | 3,962 |
| 4 | 6,231 |
| 5 | 3,103 |
| TOTAL | **19,305** |

**Supplementary Table 2. Top25 upregulated genes in the carious pulp data set.**

| **cluster** | **gene** | **p_val** | **avg_log2FC** | **pct.1** | **pct.2** | **p_val_adj** |
| --- | --- | --- | --- | --- | --- | --- |
| Immune -subcluster5 | IGKC | 2.6972E-74 | 5.55258473 | 0.782 | 0.229 | 5.1128E-70 |
|  | IGLC3 | 1.9024E-25 | 5.08625896 | 0.354 | 0.065 | 3.6062E-21 |
|  | IGHG1 | 8.2738E-38 | 4.75648189 | 0.443 | 0.074 | 1.5684E-33 |
|  | IGLC2 | 8.4419E-42 | 4.67728928 | 0.483 | 0.071 | 1.6002E-37 |
|  | IGHG4 | 5.2891E-35 | 3.64364211 | 0.361 | 0.024 | 1.0026E-30 |
|  | IGHG3 | 3.6208E-25 | 3.36232375 | 0.319 | 0.047 | 6.8636E-21 |
|  | IGHA1 | 9.5936E-16 | 3.24707783 | 0.293 | 0.082 | 1.8186E-11 |
|  | IGHG2 | 3.7373E-09 | 2.66754242 | 0.182 | 0.056 | 7.0844E-05 |
|  | BANK1 | 3.2173E-46 | 1.97209874 | 0.517 | 0.118 | 6.0988E-42 |
| nmsccs | CD74 | 6.6924E-51 | 1.83636947 | 0.5 | 0.229 | 1.2686E-46 |
| Immune -subcluster5 | CD83 | 1.5424E-78 | 1.82935524 | 0.74 | 0.162 | 2.9237E-74 |
|  | JCHAIN | 9.8022E-10 | 1.81224322 | 0.18 | 0.05 | 1.8581E-05 |
|  | BACH2 | 7.0135E-36 | 1.68899281 | 0.413 | 0.068 | 1.3295E-31 |
|  | CCL3 | 1.4453E-21 | 1.68372075 | 0.325 | 0.074 | 2.7398E-17 |
|  | SIPA1L1 | 1.0015E-83 | 1.59421381 | 0.745 | 0.156 | 1.8984E-79 |
|  | FCHSD2 | 5.1503E-76 | 1.5504018 | 0.771 | 0.235 | 9.763E-72 |
|  | PDE4D | 3.3417E-34 | 1.54939227 | 0.429 | 0.091 | 6.3345E-30 |
|  | CCL3L3 | 6.6018E-15 | 1.54481846 | 0.257 | 0.068 | 1.2514E-10 |
|  | AFF3 | 8.4533E-45 | 1.5247373 | 0.57 | 0.174 | 1.6024E-40 |
|  | HLA-DQA1 | 5.6345E-79 | 1.51836775 | 0.77 | 0.203 | 1.0681E-74 |
|  | NAMPT | 2.0969E-30 | 1.5142951 | 0.464 | 0.129 | 3.9749E-26 |
| nmsccs | HLA-DRA | 1.409E-64 | 1.51000515 | 0.433 | 0.13 | 2.6708E-60 |
| Immune -subcluster5 | NFKB1 | 8.5166E-54 | 1.4969718 | 0.543 | 0.074 | 1.6144E-49 |
| fibro | SAA1 | 5.23E-148 | 1.47113224 | 0.11 | 0.013 | 9.913E-144 |
| Immune -subcluster5 | ANKRD44 | 3.1376E-68 | 1.45879327 | 0.728 | 0.221 | 5.9477E-64 |

**Supplementary Table 3. Fraction of cells expressing *Toll-like receptors (TLR)* in the fibroblast cell cluster.**

|  | **Healthy pulp** | **Carious pulp** |
| --- | --- | --- |
| TLR1 | 0.037986 | 0.052001 |
| TLR2 | 0.000968 | 0.004066 |
| TLR3 | 0.033143 | 0.070618 |
| TLR4 | 0.032067 | 0.065055 |
| TLR5 | 0.015711 | 0.035523 |
| TLR6 | 0.007425 | 0.015836 |
| TLR7 | 0.000323 | 0.001498 |
| TLR8 | 0.000323 | 0.000428 |
| TLR10 | 0.00043 | 0.00107 |

**Supplementary Table 4. Fraction of cells expressing different Collagen types in the fibroblast cell cluster.**

|  | **Healthy pulp** | **Carious pulp** |
| --- | --- | --- |
| COL10A1 | 0.009469 | 0.012412 |
| COL11A1 | 0.044227 | 0.07661 |
| COL12A1 | 0.006564 | 0.043869 |
| COL13A1 | 0.013666 | 0.014766 |
| COL14A1 | 0.02292 | 0.020758 |
| COL15A1 | 0.020768 | 0.01926 |
| COL16A1 | 0.028624 | 0.074042 |
| COL17A1 | 0 | 0.000642 |
| COL18A1 | 0.131497 | 0.172908 |
| COL19A1 | 0.04724 | 0.113845 |
| COL1A1 | 0.360594 | 0.731436 |
| COL20A1 | 0.000323 | 0.000214 |
| COL21A1 | 0.394383 | 0.605607 |
| COL22A1 | 0.00452 | 0.013054 |
| COL23A1 | 0.002905 | 0.00856 |
| COL24A1 | 0.049177 | 0.041515 |
| COL25A1 | 0.074465 | 0.308581 |
| COL26A1 | 0.023566 | 0.027391 |
| COL27A1 | 0.046594 | 0.177616 |
| COL28A1 | 0.012483 | 0.015622 |
| COL2A1 | 0.000646 | 0.001284 |
| COL3A1 | 0.500915 | 0.661245 |
| COL4A1 | 0.024965 | 0.051359 |
| COL5A1 | 0.057463 | 0.165204 |
| COL6A1 | 0.131927 | 0.280334 |
| COL7A1 | 0.015065 | 0.044939 |
| COL8A1 | 0.132896 | 0.233897 |
| COL9A1 | 0.001399 | 0.000428 |
