# Supplementary material for: Extracellular matrix remodelling in dental pulp tissue of carious human teeth through the prism of single-cell RNA sequencing
Source: Int J Oral Sci. 2023 Aug 2;15:30. doi: 10.1038/s41368-023-00238-z (PMC10397277; doi:10.1038/s41368-023-00238-z)
Supplement: Supplementary file 2 — Supplemental Data 1 [file 41368_2023_238_MOESM2_ESM.pdf]

gProfiler_hsapiens_16-12-2022_13-26-57__intersections

source	term_name	term_id	adjusted_p_value	negative_log10_of_adjusted_p_value	term_size	query_size	intersection_size	effective_domain_size
GO:MF	signaling receptor binding	GO:0005102	2.26750144239188E-11	10.644452428006500	1555	22	16	20166
GO:MF	immunoglobulin receptor binding	GO:0034987	5.22962035944045E-10	9.281529837286550	78	22	7	20166
GO:MF	antigen binding	GO:0003823	2.04253508094633E-09	8.68983047563737	170	22	8	20166
GO:BP	positive regulation of leukocyte activation	GO:0002696	6.9055979597383E-09	8.160798709645660	441	21	10	21092
GO:BP	positive regulation of cell activation	GO:0050867	9.82191956374581E-09	8.007803626828770	457	21	10	21092
GO:BP	regulation of leukocyte activation	GO:0002694	1.09176576497239E-08	7.96187052819885	657	21	11	21092
GO:BP	immune response	GO:0006955	1.172052603629E-08	7.931052896037100	1913	21	15	21092
GO:BP	regulation of B cell activation	GO:0050864	1.87728100064647E-08	7.726470715180380	202	21	8	21092
GO:BP	positive regulation of immune system process	GO:0002684	2.30116533123226E-08	7.6380521775667300	954	21	12	21092
GO:BP	regulation of cell activation	GO:0050865	2.59625398741883E-08	7.585652823451480	712	21	11	21092
GO:BP	adaptive immune response based on somatic recombination of immune receptors built from immunoglobulin superfamily domains	GO:0002460	4.2236564178454E-08	7.374311417629260	357	21	9	21092
GO:BP	B cell receptor signaling pathway	GO:0050853	4.63813206209727E-08	7.3336568898024800	128	21	7	21092
GO:BP	regulation of lymphocyte activation	GO:0051249	7.9267647982583E-08	7.100904027934260	565	21	10	21092
GO:BP	antigen receptor-mediated signaling pathway	GO:0050851	8.51849054871005E-08	7.0696373541115800	244	21	8	21092
GO:BP	positive regulation of lymphocyte activation	GO:0051251	1.01617259719688E-07	6.993032520748940	394	21	9	21092
GO:BP	positive regulation of immune response	GO:0050778	1.23181394970247E-07	6.909454882041100	591	21	10	21092
GO:BP	positive regulation of B cell activation	GO:0050871	1.71531199046776E-07	6.765656876545150	154	21	7	21092
GO:BP	cell activation	GO:0001775	1.79083549060133E-07	6.746944307398050	1138	21	12	21092
GO:BP	immune system process	GO:0002376	1.9676498675665E-07	6.70605217933566	2842	21	16	21092
GO:BP	leukocyte mediated immunity	GO:0002443	3.05106561572809E-07	6.515548452389650	446	21	9	21092
GO:BP	immune response-activating cell surface receptor signaling pathway	GO:0002429	4.6463420099331E-07	6.332888825624910	302	21	8	21092
GO:BP	immune response-activating signal transduction	GO:0002757	4.6463420099331E-07	6.332888825624910	302	21	8	21092
GO:BP	humoral immune response	GO:0006959	6.99328104341606E-07	6.1553190181415500	318	21	8	21092
GO:BP	adaptive immune response	GO:0002250	8.5616827180115E-07	6.067440870448220	721	21	10	21092
GO:BP	leukocyte activation	GO:0045321	9.27678892533402E-07	6.032602324787200	995	21	11	21092
GO:BP	phagocytosis, recognition	GO:0006910	9.39838296382324E-07	6.026946862389620	102	21	6	21092
GO:BP	immune response-regulating cell surface receptor signaling pathway	GO:0002768	9.60025885585988E-07	6.017717056735860	331	21	8	21092
GO:BP	immunoglobulin mediated immune response	GO:0016064	0.0000010374914455063100	5.984015475516810	199	21	7	21092
GO:BP	B cell mediated immunity	GO:0019724	0.0000011518775240040300	5.938593695805970	202	21	7	21092
GO:BP	complement activation, classical pathway	GO:0006958	0.0000012575349923100800	5.9004799213768200	107	21	6	21092
GO:BP	B cell activation	GO:0042113	0.0000013936679398030200	5.85584069043461	347	21	8	21092
GO:BP	lymphocyte mediated immunity	GO:0002449	0.0000014256687616428500	5.845981366280060	348	21	8	21092
GO:BP	response to bacterium	GO:0009617	0.000001427157247558330	5.845528172656580	760	21	10	21092
GO:BP	defense response	GO:0006952	0.0000014379398092203200	5.842259292722660	1742	21	13	21092
GO:BP	humoral immune response mediated by circulating immunoglobulin	GO:0002455	0.000002521553178977550	5.598331868237360	120	21	6	21092
GO:BP	activation of immune response	GO:0002253	0.0000032240838914829600	5.4915936662670300	386	21	8	21092
GO:BP	phagocytosis, engulfment	GO:0006911	0.0000033874595139342800	5.470125886665140	126	21	6	21092
GO:BP	lymphocyte activation	GO:0046649	0.000003503983625091860	5.455437932055570	834	21	10	21092
GO:BP	regulation of immune system process	GO:0002682	0.00000391927678145537	5.406794065326640	1488	21	12	21092
GO:BP	complement activation	GO:0006956	0.000004091528728196300	5.388114395128650	130	21	6	21092
GO:BP	response to other organism	GO:0051707	0.000004740197755032500	5.324203539733540	1513	21	12	21092
GO:BP	response to external biotic stimulus	GO:0043207	0.000004848525893666710	5.314390280687730	1516	21	12	21092
GO:BP	plasma membrane invagination	GO:0099024	0.000005138162431802200	5.2891921705795300	135	21	6	21092
GO:BP	response to biotic stimulus	GO:0009607	0.00000642913189879137	5.191847664247900	1554	21	12	21092
GO:BP	membrane invagination	GO:0010324	0.000006968153646712230	5.1568822817685300	142	21	6	21092
GO:BP	regulation of immune response	GO:0050776	0.000007216817871794120	5.141654254695700	899	21	10	21092
GO:BP	innate immune response	GO:0045087	0.00000863930979335993	5.063520952533690	916	21	10	21092
GO:BP	immune effector process	GO:0002252	0.000012360234974199700	4.9079732730151400	680	21	9	21092
GO:BP	biological process involved in interspecies interaction between organisms	GO:0044419	0.000015068447900033400	4.821931479149560	1675	21	12	21092
GO:BP	immune response-regulating signaling pathway	GO:0002764	0.0000211732555202971	4.674212361357030	491	21	8	21092
GO:BP	positive regulation of response to stimulus	GO:0048584	0.000027339403349325200	4.563210967636640	2214	21	13	21092
GO:BP	defense response to bacterium	GO:0042742	0.000074366009431433330	4.128625522646000	369	21	7	21092
GO:BP	defense response to other organism	GO:0098542	0.000084109356423057500	4.075155690089310	1163	21	10	21092
GO:BP	cell recognition	GO:0008037	0.00012107067297550600	3.9169610435404900	229	21	6	21092
GO:BP	positive regulation of biological process	GO:0048518	0.000295755185023197	3.5290676328125500	6309	21	18	21092
GO:BP	regulation of response to stimulus	GO:0048583	0.00036650137661875300	3.435924389762810	3970	21	15	21092
GO:BP	response to external stimulus	GO:0009605	0.0004912236745281790	3.3087207105371200	2814	21	13	21092
GO:BP	cell surface receptor signaling pathway	GO:0007166	0.0004953953835858690	3.3050480447677900	2816	21	13	21092
GO:BP	phagocytosis	GO:0006909	0.0007350377784248230	3.1336903390929500	311	21	6	21092
GO:BP	response to stimulus	GO:0050896	0.0008225961389373370	3.084813333292860	9000	21	20	21092
GO:BP	endocytosis	GO:0006897	0.0035397687231559600	2.4510251124200800	654	21	7	21092
GO:BP	positive regulation of cellular process	GO:0048522	0.005407183350547470	2.2670289038142500	5641	21	16	21092
GO:BP	neutrophil chemotaxis	GO:0030593	0.005627758402761220	2.2496645549117400	106	21	4	21092
GO:BP	regulation of cytokine production	GO:0001817	0.007529730096553210	2.1232205908216600	733	21	7	21092
GO:BP	cytokine production	GO:0001816	0.007945356968829390	2.0998865860450000	739	21	7	21092
GO:BP	neutrophil migration	GO:1990266	0.013060803181480400	1.8840301150548700	131	21	4	21092
GO:BP	granulocyte chemotaxis	GO:0071621	0.013060803181480400	1.8840301150548700	131	21	4	21092
GO:BP	regulation of biological process	GO:0050789	0.02215361463771800	1.6545554031140300	12361	21	21	21092
GO:BP	response to stress	GO:0006950	0.02463974319126670	1.6083638229360600	3938	21	13	21092
GO:BP	membrane organization	GO:0061024	0.024913634480500000	1.6035629115768400	880	21	7	21092
GO:BP	granulocyte migration	GO:0097530	0.028766722173001000	1.5411096210181900	160	21	4	21092
GO:BP	signaling	GO:0023052	0.03965272783068730	1.4017269308920200	6492	21	16	21092
GO:BP	cell communication	GO:0007154	0.04500020272006940	1.346785529780010	6551	21	16	21092
GO:BP	regulation of cellular process	GO:0050794	0.0484422616385589	1.3147755890174800	11130	21	20	21092
GO:CC	IgG immunoglobulin complex	GO:0071735	1.22867145685204E-13	12.910564230662100	5	22	5	21690
GO:CC	immunoglobulin complex, circulating	GO:0042571	1.3537198467344E-10	9.868471203897710	75	22	7	21690
GO:CC	blood microparticle	GO:0072562	1.01021941817653E-08	7.995584287847160	137	22	7	21690
GO:CC	immunoglobulin complex	GO:0019814	3.74971301447594E-08	7.426001969871990	165	22	7	21690
GO:CC	external side of plasma membrane	GO:0009897	0.0000017405899576502400	5.75930352647247	459	22	8	21690
GO:CC	side of membrane	GO:0098552	0.0000018102051678045000	5.742272199608330	681	22	9	21690
GO:CC	IgA immunoglobulin complex	GO:0071745	0.0000033725797140366300	5.472037776583530	6	22	3	21690
GO:CC	cell surface	GO:0009986	0.000036490760928359400	4.437817080358510	966	22	9	21690
GO:CC	extracellular exosome	GO:0070062	0.00033942775541812900	3.4692526477812000	2108	22	11	21690
GO:CC	extracellular vesicle	GO:1903561	0.0003799062056479250	3.420323612324090	2132	22	11	21690
GO:CC	extracellular membrane-bounded organelle	GO:0065010	0.00038168200725268100	3.4182983125032300	2133	22	11	21690
GO:CC	extracellular organelle	GO:0043230	0.00038168200725268100	3.4182983125032300	2133	22	11	21690
GO:CC	secretory dimeric IgA immunoglobulin complex	GO:0071752	0.0005493286909810060	3.2601677175464000	3	22	2	21690
GO:CC	dimeric IgA immunoglobulin complex	GO:0071750	0.0005493286909810060	3.2601677175464000	3	22	2	21690
GO:CC	extracellular space	GO:0005615	0.0006907485220281170	3.160680035656070	3368	22	13	21690
GO:CC	monomeric IgA immunoglobulin complex	GO:0071748	0.0010979819787380200	2.9594047879358900	4	22	2	21690
GO:CC	IgM immunoglobulin complex	GO:0071753	0.0010979819787380200	2.9594047879358900	4	22	2	21690
GO:CC	extracellular region	GO:0005576	0.0016814247241685100	2.7743225708768600	4302	22	14	21690
GO:CC	secretory IgA immunoglobulin complex	GO:0071751	0.001828845032169930	2.7378230930609300	5	22	2	21690
GO:CC	IgA immunoglobulin complex, circulating	GO:0071746	0.001828845032169930	2.7378230930609300	5	22	2	21690
GO:CC	polymeric IgA immunoglobulin complex	GO:0071749	0.001828845032169930	2.7378230930609300	5	22	2	21690
GO:CC	vesicle	GO:0031982	0.004514200578379840	2.3454191479281200	3973	22	13	21690
GO:CC	MHC class II protein complex	GO:0042613	0.02468948566096570	1.6074879573313800	17	22	2	21690
GO:CC	MHC protein complex	GO:0042611	0.049890069071209700	1.3019858947935400	24	22	2	21690
KEGG	Rheumatoid arthritis	KEGG:05323	0.0036113231754368000	2.442333645046280	88	8	3	8064
KEGG	Chagas disease	KEGG:05142	0.005451037263128000	2.2635208491380400	101	8	3	8064
KEGG	Toll-like receptor signaling pathway	KEGG:04620	0.005613590041675640	2.250759306902710	102	8	3	8064
KEGG	Tuberculosis	KEGG:05152	0.027737316834542100	1.5569355526467400	175	8	3	8064
KEGG	Chemokine signaling pathway	KEGG:04062	0.03529685175987670	1.4522640290112400	190	8	3	8064
KEGG	Lipid and atherosclerosis	KEGG:05417	0.0499546758995051	1.301423854338710	214	8	3	8064
REAC	Classical antibody-mediated complement activation	REAC:R-HSA-	0.000006976291430464630	5.156375385300410	61	16	5	10461
REAC	FCGR activation	REAC:R-HSA-	0.00001126544188040360	4.9482517686576700	67	16	5	10461
REAC	Creation of C4 and C2 activators	REAC:R-HSA-	0.000012148826865871800	4.91546565706785	68	16	5	10461
REAC	Initial triggering of complement	REAC:R-HSA-	0.00002138043322191340	4.669983499128160	76	16	5	10461
REAC	Role of phospholipids in phagocytosis	REAC:R-HSA-	0.000029520896720181500	4.529870454616210	81	16	5	10461
REAC	FCGR3A-mediated IL10 synthesis	REAC:R-HSA-	0.00005925086862085290	4.227305278491220	93	16	5	10461
REAC	Binding and Uptake of Ligands by Scavenger Receptors	REAC:R-HSA-	0.00007321431871310540	4.135403974543220	97	16	5	10461
REAC	Regulation of Complement cascade	REAC:R-HSA-	0.00008966878735557430	4.047358703424330	101	16	5	10461
REAC	Complement cascade	REAC:R-HSA-	0.000143815524263021	3.842194231214180	111	16	5	10461
REAC	Parasite infection	REAC:R-HSA-	0.00015722741474939300	3.8034717265133400	113	16	5	10461
REAC	FCGR3A-mediated phagocytosis	REAC:R-HSA-	0.00015722741474939300	3.8034717265133400	113	16	5	10461
REAC	Leishmania phagocytosis	REAC:R-HSA-	0.00015722741474939300	3.8034717265133400	113	16	5	10461
REAC	Regulation of actin dynamics for phagocytic cup formation	REAC:R-HSA-	0.00017918307610285500	3.7467030119854700	116	16	5	10461
REAC	Fcgamma receptor (FCGR) dependent phagocytosis	REAC:R-HSA-	0.000456053510722036	3.3409841967042900	140	16	5	10461
REAC	Scavenging of heme from plasma	REAC:R-HSA-	0.0008741054170048670	3.058436188346430	69	16	4	10461
REAC	Leishmania infection	REAC:R-HSA-	0.00105610656383979	2.9762922581728300	304	16	6	10461
REAC	Cell surface interactions at the vascular wall	REAC:R-HSA-	0.002047596099688850	2.6887557063757600	190	16	5	10461
REAC	Leishmania parasite growth and survival	REAC:R-HSA-	0.004273164584218070	2.3692503796749200	221	16	5	10461
REAC	Anti-inflammatory response favouring Leishmania parasite infection	REAC:R-HSA-	0.004273164584218070	2.3692503796749200	221	16	5	10461
REAC	Immune System	REAC:R-HSA-	0.007305583102728660	2.136345114679590	2039	16	11	10461
REAC	Signaling by Interleukins	REAC:R-HSA-	0.011307913648726100	1.9466175165978800	460	16	6	10461
REAC	Cytokine Signaling in Immune system	REAC:R-HSA-	0.011933492821615100	1.9232324237949600	704	16	7	10461
WP	Macrophage markers	WP:WP4146	0.005449540709434400	2.2636400987809000	9	10	2	7827
WP	NAD metabolism, sirtuins and aging	WP:WP3630	0.008314340992584660	2.0801721679268600	11	10	2	7827
WP	Overview of proinflammatory and profibrotic mediators	WP:WP5095	0.04768633720510710	1.3216060345110000	127	10	3	7827
HPA	tonsil; non-germinal center cells[≥Medium]	HPA:0600442	0.00007115980081102900	4.147765276254110	4174	15	15	10976
HPA	tonsil; non-germinal center cells[≥Low]	HPA:0600441	0.020327234861852500	1.6919216949435800	6082	15	15	10976
CORUM	AKAP250-PKA-PDE4D complex	CORUM:518	0.049986628576846000	1.3011461539025000	2	1	1	3385

intersections
IGLC3,IGHG1,IGLC2,IGHG4,IGHA1,IGHG2,BANK1,CD74,JCHAIN,CCL3,SIPA1L1,PDE4D,CCL3L3,NAMPT,HLA-DRA,SAA1
IGLC3,IGHG1,IGLC2,IGHG4,IGHA1,IGHG2,JCHAIN
IGLC3,IGHG1,IGLC2,IGHG4,IGHA1,IGHG2,JCHAIN,HLA-DRA
IGLC3,IGHG1,IGLC2,IGHG4,IGHA1,IGHG2,CD74,CD83,CCL3,HLA-DRA
IGLC3,IGHG1,IGLC2,IGHG4,IGHA1,IGHG2,CD74,CD83,CCL3,HLA-DRA
IGLC3,IGHG1,IGLC2,IGHG4,IGHA1,IGHG2,BANK1,CD74,CD83,CCL3,HLA-DRA
IGLC3,IGHG1,IGLC2,IGHG4,IGHA1,IGHG2,BANK1,CD74,CD83,JCHAIN,BACH2,CCL3,PDE4D,CCL3L3,HLA-DRA
IGLC3,IGHG1,IGLC2,IGHG4,IGHA1,IGHG2,BANK1,CD74
IGLC3,IGHG1,IGLC2,IGHG4,IGHA1,IGHG2,BANK1,CD74,CD83,CCL3,PDE4D,HLA-DRA
IGLC3,IGHG1,IGLC2,IGHG4,IGHA1,IGHG2,BANK1,CD74,CD83,CCL3,HLA-DRA
IGLC3,IGHG1,IGLC2,IGHG4,IGHA1,IGHG2,CD74,BACH2,HLA-DRA
IGLC3,IGHG1,IGLC2,IGHG4,IGHA1,IGHG2,BANK1
IGLC3,IGHG1,IGLC2,IGHG4,IGHA1,IGHG2,BANK1,CD74,CD83,HLA-DRA
IGLC3,IGHG1,IGLC2,IGHG4,IGHA1,IGHG2,BANK1,PDE4D
IGLC3,IGHG1,IGLC2,IGHG4,IGHA1,IGHG2,CD74,CD83,HLA-DRA
IGLC3,IGHG1,IGLC2,IGHG4,IGHA1,IGHG2,BANK1,CD74,PDE4D,HLA-DRA
IGLC3,IGHG1,IGLC2,IGHG4,IGHA1,IGHG2,CD74
IGLC3,IGHG1,IGLC2,IGHG4,IGHA1,IGHG2,BANK1,CD74,CD83,CCL3,HLA-DRA,SAA1
IGLC3,IGHG1,IGLC2,IGHG4,IGHA1,IGHG2,BANK1,CD74,CD83,JCHAIN,BACH2,CCL3,PDE4D,CCL3L3,HLA-DRA,SAA1
IGLC3,IGHG1,IGLC2,IGHG4,IGHA1,IGHG2,CD74,CCL3,HLA-DRA
IGLC3,IGHG1,IGLC2,IGHG4,IGHA1,IGHG2,BANK1,PDE4D
IGLC3,IGHG1,IGLC2,IGHG4,IGHA1,IGHG2,BANK1,PDE4D
IGLC3,IGHG1,IGLC2,IGHG4,IGHA1,IGHG2,CD83,JCHAIN
IGLC3,IGHG1,IGLC2,IGHG4,IGHA1,IGHG2,CD74,JCHAIN,BACH2,HLA-DRA
IGLC3,IGHG1,IGLC2,IGHG4,IGHA1,IGHG2,BANK1,CD74,CD83,CCL3,HLA-DRA
IGLC3,IGHG1,IGLC2,IGHG4,IGHA1,IGHG2
IGLC3,IGHG1,IGLC2,IGHG4,IGHA1,IGHG2,BANK1,PDE4D
IGLC3,IGHG1,IGLC2,IGHG4,IGHA1,IGHG2,CD74
IGLC3,IGHG1,IGLC2,IGHG4,IGHA1,IGHG2,CD74
IGLC3,IGHG1,IGLC2,IGHG4,IGHA1,IGHG2
IGLC3,IGHG1,IGLC2,IGHG4,IGHA1,IGHG2,BANK1,CD74
IGLC3,IGHG1,IGLC2,IGHG4,IGHA1,IGHG2,CD74,HLA-DRA
IGLC3,IGHG1,IGLC2,IGHG4,IGHA1,IGHG2,BANK1,JCHAIN,CCL3,NFKB1
IGLC3,IGHG1,IGLC2,IGHG4,IGHA1,IGHG2,CD74,CD83,JCHAIN,CCL3,CCL3L3,NFKB1,SAA1
IGLC3,IGHG1,IGLC2,IGHG4,IGHA1,IGHG2
IGLC3,IGHG1,IGLC2,IGHG4,IGHA1,IGHG2,BANK1,PDE4D
IGLC3,IGHG1,IGLC2,IGHG4,IGHA1,IGHG2
IGLC3,IGHG1,IGLC2,IGHG4,IGHA1,IGHG2,BANK1,CD74,CD83,HLA-DRA
IGLC3,IGHG1,IGLC2,IGHG4,IGHA1,IGHG2,BANK1,CD74,CD83,CCL3,PDE4D,HLA-DRA
IGLC3,IGHG1,IGLC2,IGHG4,IGHA1,IGHG2
IGLC3,IGHG1,IGLC2,IGHG4,IGHA1,IGHG2,BANK1,CD74,JCHAIN,CCL3,CCL3L3,NFKB1
IGLC3,IGHG1,IGLC2,IGHG4,IGHA1,IGHG2,BANK1,CD74,JCHAIN,CCL3,CCL3L3,NFKB1
IGLC3,IGHG1,IGLC2,IGHG4,IGHA1,IGHG2
IGLC3,IGHG1,IGLC2,IGHG4,IGHA1,IGHG2,BANK1,CD74,JCHAIN,CCL3,CCL3L3,NFKB1
IGLC3,IGHG1,IGLC2,IGHG4,IGHA1,IGHG2
IGLC3,IGHG1,IGLC2,IGHG4,IGHA1,IGHG2,BANK1,CD74,PDE4D,HLA-DRA
IGLC3,IGHG1,IGLC2,IGHG4,IGHA1,IGHG2,CD74,JCHAIN,CCL3,CCL3L3
IGLC3,IGHG1,IGLC2,IGHG4,IGHA1,IGHG2,CD74,CCL3,HLA-DRA
IGLC3,IGHG1,IGLC2,IGHG4,IGHA1,IGHG2,BANK1,CD74,JCHAIN,CCL3,CCL3L3,NFKB1
IGLC3,IGHG1,IGLC2,IGHG4,IGHA1,IGHG2,BANK1,PDE4D
IGLC3,IGHG1,IGLC2,IGHG4,IGHA1,IGHG2,BANK1,CD74,CCL3,PDE4D,CCL3L3,HLA-DRA,NFKB1
IGLC3,IGHG1,IGLC2,IGHG4,IGHA1,IGHG2,JCHAIN
IGLC3,IGHG1,IGLC2,IGHG4,IGHA1,IGHG2,CD74,JCHAIN,CCL3,CCL3L3
IGLC3,IGHG1,IGLC2,IGHG4,IGHA1,IGHG2
IGLC3,IGHG1,IGLC2,IGHG4,IGHA1,IGHG2,BANK1,CD74,CD83,JCHAIN,CCL3,FCHSD2,PDE4D,CCL3L3,NAMPT,HLA-DRA,NFKB1,SAA1
IGLC3,IGHG1,IGLC2,IGHG4,IGHA1,IGHG2,BANK1,CD74,CCL3,SIPA1L1,PDE4D,CCL3L3,HLA-DRA,NFKB1,SAA1
IGLC3,IGHG1,IGLC2,IGHG4,IGHA1,IGHG2,BANK1,CD74,JCHAIN,CCL3,CCL3L3,NFKB1,SAA1
IGLC3,IGHG1,IGLC2,IGHG4,IGHA1,IGHG2,BANK1,CD74,CCL3,SIPA1L1,PDE4D,CCL3L3,NFKB1
IGLC3,IGHG1,IGLC2,IGHG4,IGHA1,IGHG2
IGLC3,IGHG1,IGLC2,IGHG4,IGHA1,IGHG2,BANK1,CD74,CD83,JCHAIN,BACH2,CCL3,SIPA1L1,PDE4D,CCL3L3,AFF3,NAMPT,HLA-DRA,NFKB1,SAA1
IGLC3,IGHG1,IGLC2,IGHG4,IGHA1,IGHG2,FCHSD2
IGLC3,IGHG1,IGLC2,IGHG4,IGHA1,IGHG2,BANK1,CD74,CD83,CCL3,FCHSD2,CCL3L3,NAMPT,HLA-DRA,NFKB1,SAA1
CD74,CCL3,CCL3L3,SAA1
BANK1,CD74,CD83,CCL3,PDE4D,NFKB1,SAA1
BANK1,CD74,CD83,CCL3,PDE4D,NFKB1,SAA1
CD74,CCL3,CCL3L3,SAA1
CD74,CCL3,CCL3L3,SAA1
IGLC3,IGHG1,IGLC2,IGHG4,IGHA1,IGHG2,BANK1,CD74,CD83,JCHAIN,BACH2,CCL3,SIPA1L1,FCHSD2,PDE4D,CCL3L3,AFF3,NAMPT,HLA-DRA,NFK[illegible]
IGLC3,IGHG1,IGLC2,IGHG4,IGHA1,IGHG2,CD74,CD83,JCHAIN,CCL3,CCL3L3,NFKB1,SAA1
IGLC3,IGHG1,IGLC2,IGHG4,IGHA1,IGHG2,FCHSD2
CD74,CCL3,CCL3L3,SAA1
IGLC3,IGHG1,IGLC2,IGHG4,IGHA1,IGHG2,BANK1,CD74,CD83,CCL3,SIPA1L1,FCHSD2,PDE4D,CCL3L3,NAMPT,NFKB1
IGLC3,IGHG1,IGLC2,IGHG4,IGHA1,IGHG2,BANK1,CD74,CD83,CCL3,SIPA1L1,FCHSD2,PDE4D,CCL3L3,NAMPT,NFKB1
IGLC3,IGHG1,IGLC2,IGHG4,IGHA1,IGHG2,BANK1,CD74,CD83,BACH2,CCL3,SIPA1L1,FCHSD2,PDE4D,CCL3L3,AFF3,NAMPT,HLA-DRA,NFKB1,SAA1
IGLC3,IGHG1,IGHG4,IGHA1,IGHG2
IGLC3,IGHG1,IGLC2,IGHG4,IGHA1,IGHG2,JCHAIN
IGLC3,IGHG1,IGLC2,IGHG4,IGHA1,IGHG2,JCHAIN
IGLC3,IGHG1,IGLC2,IGHG4,IGHA1,IGHG2,JCHAIN
IGLC3,IGHG1,IGLC2,IGHG4,IGHA1,IGHG2,CD74,CD83
IGLC3,IGHG1,IGLC2,IGHG4,IGHA1,IGHG2,CD74,CD83,HLA-DRA
IGLC3,IGHA1,JCHAIN
IGLC3,IGHG1,IGLC2,IGHG4,IGHA1,IGHG2,CD74,CD83,HLA-DRA
IGLC3,IGHG1,IGLC2,IGHG4,IGHA1,IGHG2,CD74,JCHAIN,NAMPT,HLA-DRA,SAA1
IGLC3,IGHG1,IGLC2,IGHG4,IGHA1,IGHG2,CD74,JCHAIN,NAMPT,HLA-DRA,SAA1
IGLC3,IGHG1,IGLC2,IGHG4,IGHA1,IGHG2,CD74,JCHAIN,NAMPT,HLA-DRA,SAA1
IGLC3,IGHG1,IGLC2,IGHG4,IGHA1,IGHG2,CD74,JCHAIN,NAMPT,HLA-DRA,SAA1
IGHA1,JCHAIN
IGHA1,JCHAIN
IGLC3,IGHG1,IGLC2,IGHG4,IGHA1,IGHG2,CD74,JCHAIN,CCL3,CCL3L3,NAMPT,HLA-DRA,SAA1
IGHA1,JCHAIN
IGLC3,JCHAIN
IGLC3,IGHG1,IGLC2,IGHG4,IGHA1,IGHG2,CD74,JCHAIN,CCL3,CCL3L3,NAMPT,HLA-DRA,NFKB1,SAA1
IGHA1,JCHAIN
IGHA1,JCHAIN
IGHA1,JCHAIN
IGLC3,IGHG1,IGLC2,IGHG4,IGHA1,IGHG2,CD74,JCHAIN,FCHSD2,NAMPT,HLA-DRA,NFKB1,SAA1
CD74,HLA-DRA
CD74,HLA-DRA
CCL3,CCL3L3,HLA-DRA
CCL3,CCL3L3,NFKB1
CCL3,CCL3L3,NFKB1
CD74,HLA-DRA,NFKB1
CCL3,CCL3L3,NFKB1
CCL3,CCL3L3,NFKB1
IGLC3,IGHG1,IGLC2,IGHG4,IGHG2
IGLC3,IGHG1,IGLC2,IGHG4,IGHG2
IGLC3,IGHG1,IGLC2,IGHG4,IGHG2
IGLC3,IGHG1,IGLC2,IGHG4,IGHG2
IGLC3,IGHG1,IGLC2,IGHG4,IGHG2
IGLC3,IGHG1,IGLC2,IGHG4,IGHG2
IGLC3,IGLC2,IGHA1,JCHAIN,SAA1
IGLC3,IGHG1,IGLC2,IGHG4,IGHG2
IGLC3,IGHG1,IGLC2,IGHG4,IGHG2
IGLC3,IGHG1,IGLC2,IGHG4,IGHG2
IGLC3,IGHG1,IGLC2,IGHG4,IGHG2
IGLC3,IGHG1,IGLC2,IGHG4,IGHG2
IGLC3,IGHG1,IGLC2,IGHG4,IGHG2
IGLC3,IGHG1,IGLC2,IGHG4,IGHG2
IGLC3,IGLC2,IGHA1,JCHAIN
IGLC3,IGHG1,IGLC2,IGHG4,IGHG2,NFKB1
IGLC3,IGLC2,IGHA1,CD74,JCHAIN
IGLC3,IGHG1,IGLC2,IGHG4,IGHG2
IGLC3,IGHG1,IGLC2,IGHG4,IGHG2
IGLC3,IGHG1,IGLC2,IGHG4,IGHG2,CD74,CCL3,CCL3L3,HLA-DRA,NFKB1,SAA1
IGHG1,IGHG4,CCL3,CCL3L3,NFKB1,SAA1
IGHG1,IGHG4,CCL3,CCL3L3,HLA-DRA,NFKB1,SAA1
CD74,CD83
NAMPT,NFKB1
CCL3,CCL3L3,NFKB1
IGHG1,IGHG4,IGHA1,IGHG2,BANK1,CD74,CD83,JCHAIN,BACH2,SIPA1L1,AFF3,NAMPT,HLA-DRA,NFKB1,ANKRD44
IGHG1,IGHG4,IGHA1,IGHG2,BANK1,CD74,CD83,JCHAIN,BACH2,SIPA1L1,AFF3,NAMPT,HLA-DRA,NFKB1,ANKRD44
PDE4D
