# Supplementary figures and images for: Extracellular matrix remodelling in dental pulp tissue of carious human teeth through the prism of single-cell RNA sequencing
Source: Int J Oral Sci. 2023 Aug 2;15:30. doi: 10.1038/s41368-023-00238-z (PMC10397277; doi:10.1038/s41368-023-00238-z)

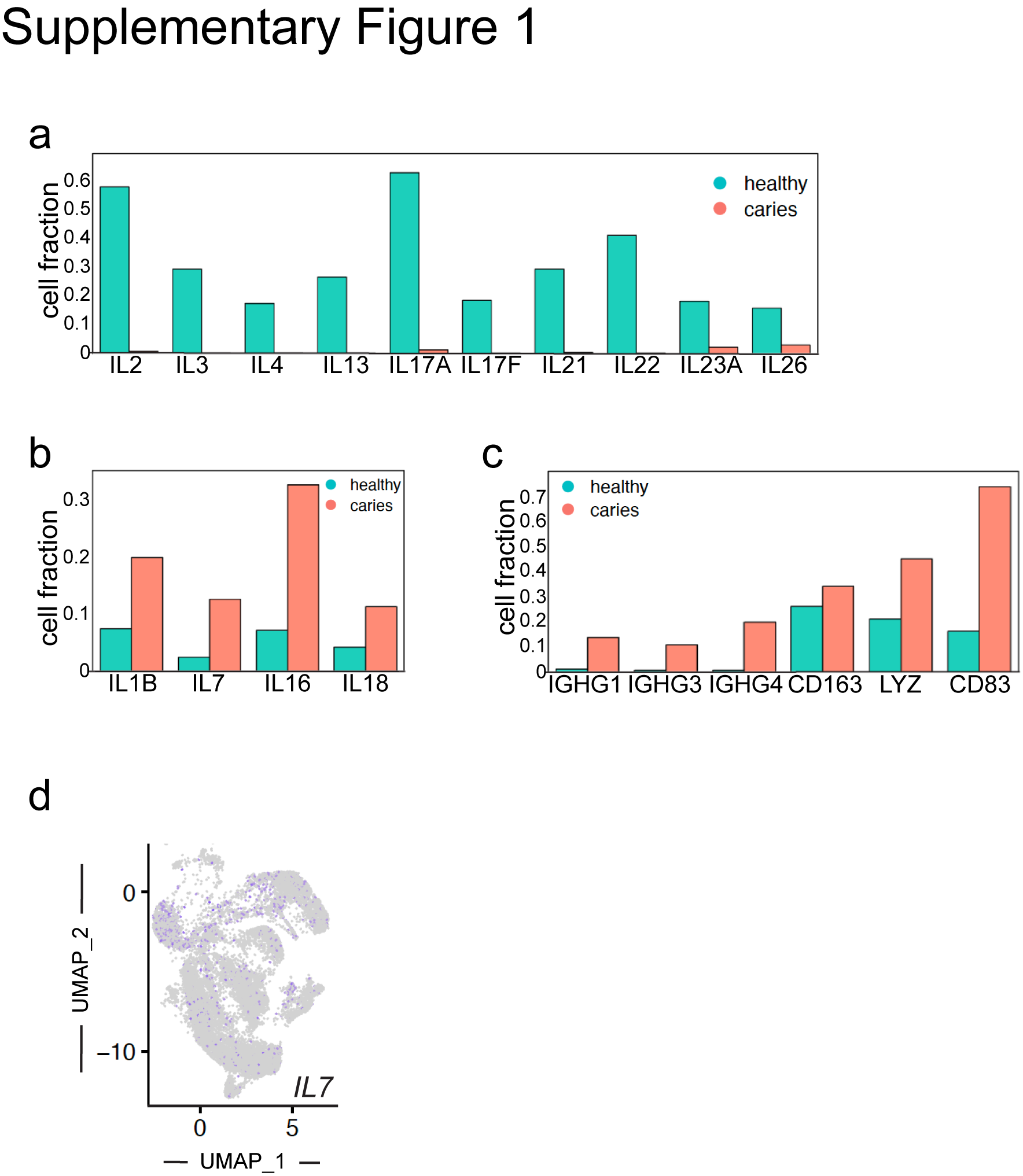

Supplement: Supplementary file 3 — Supplemental Figure 1 [file 41368_2023_238_MOESM3_ESM.tif]

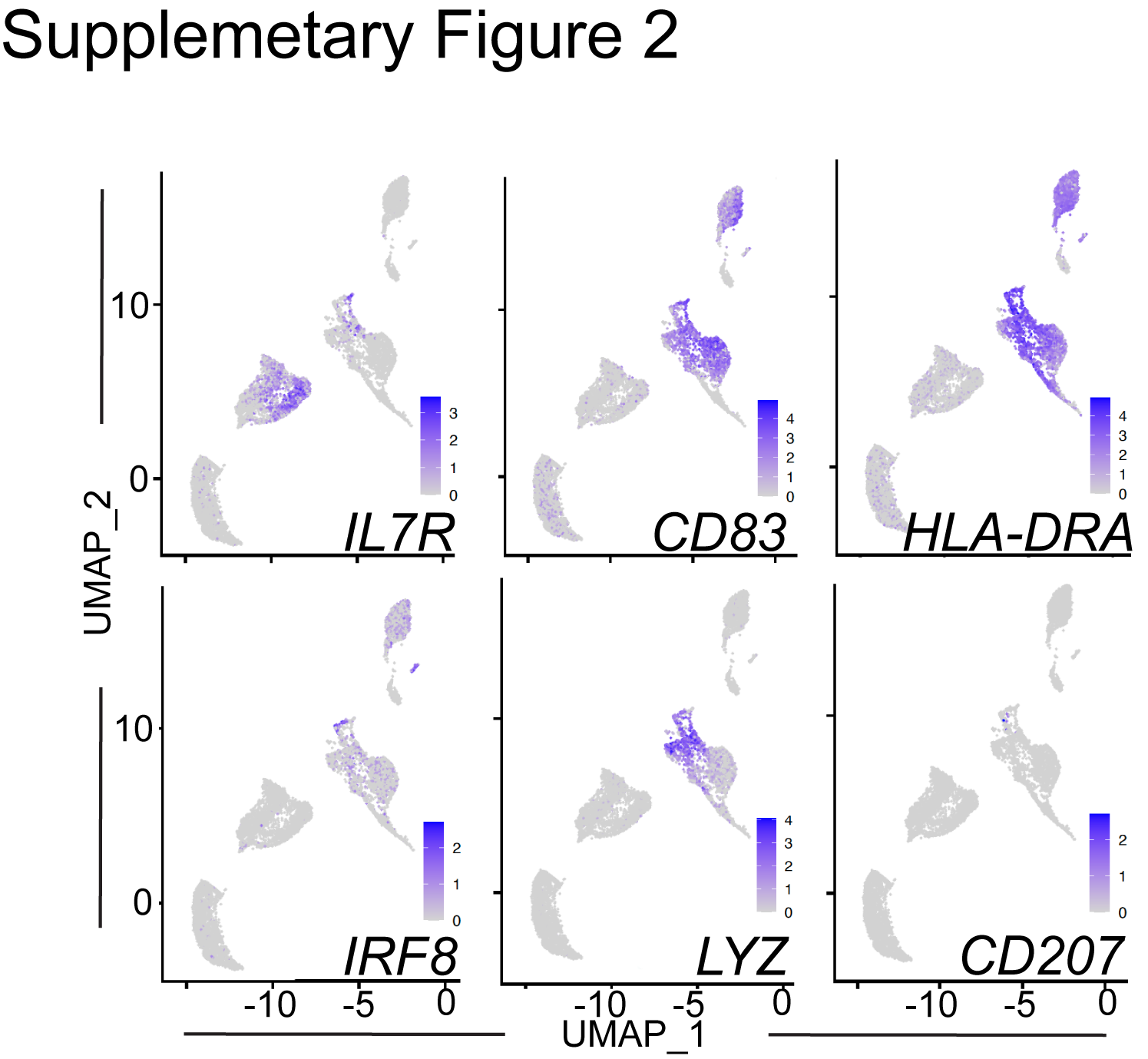

Supplement: Supplementary file 4 — Supplemental Figure 2 [file 41368_2023_238_MOESM4_ESM.tif]

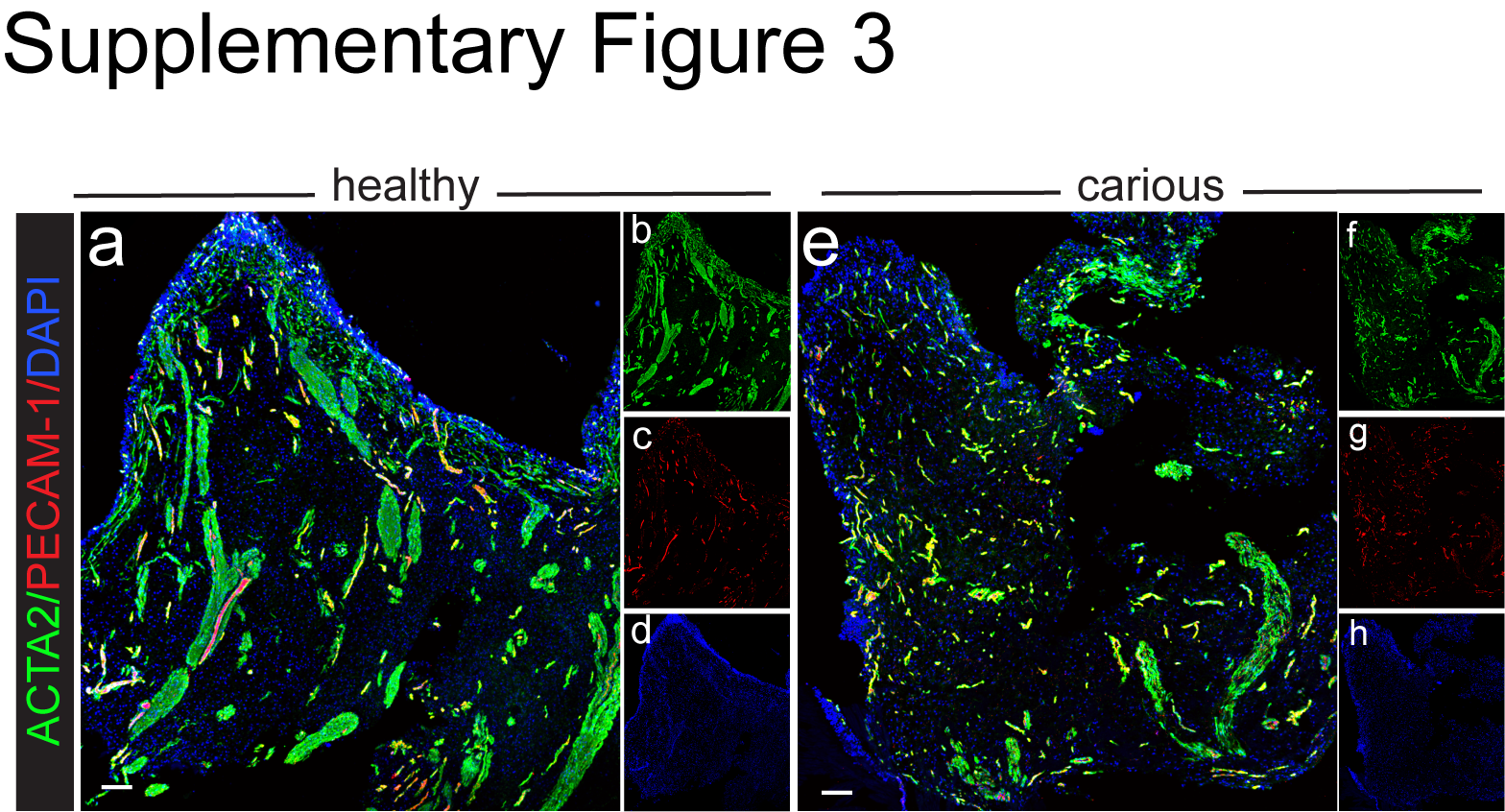

Supplement: Supplementary file 5 — Supplemental Figure 3 [file 41368_2023_238_MOESM5_ESM.tif]
