# Supplementary material for: Extracellular matrix remodelling in dental pulp tissue of carious human teeth through the prism of single-cell RNA sequencing
Source: Int J Oral Sci. 2023 Aug 2;15:30. doi: 10.1038/s41368-023-00238-z (PMC10397277; doi:10.1038/s41368-023-00238-z)
Supplement: Supplementary file 8 — Supplemental Data 3 [file 41368_2023_238_MOESM8_ESM.docx]

**Supplementary Figure 1. Analysis of immune cell related genes. a, b)** Fractions of cell populations in the immune cluster expressing IL genes, in healthy (green) and carious (red) pulps. Cell fractions of specific cell populations found mainly in the immune cluster of healthy dental pulp (a), or expanded in the carious pulps (b). **c)** Cell fractions characterised by the expression of molecular markers also upregulated in the immune cluster of carious pulps. **d)**  UMAP visualisation of the IL-7 distribution in the fibroblast subclusters of healthy and carious pulps.

**Supplementary Figure 2. Analysis of the immune subclusters.**  Feature UMAP plot representation of molecular markers shared between the different subclusters of immune cells in pulp tissues of healthy and carious teeth.

**Supplementary Figure 3. Analysis of ACTA2 and PECAM-1 expression in the healthy and carious pulps.** Immunostaining of the cryosections of healthy (a-d) and carious (e-h) pulps for ACTA2 (green) and PECAM-1 (red). Nuclei are marked by DAPI staining (blue). Individual channel images for ACTA2 (b, f), PECAM-1 (c, g) and DAPI (d, h) expression from a and e, respectively. Scale bars represent 100μm.
